# High‐quality genome of allotetraploid *Avena barbata* provides insights into the origin and evolution of B subgenome in *Avena*

**DOI:** 10.1111/jipb.13902

**Published:** 2025-04-14

**Authors:** Qiang He, Yao Xiao, Tao Li, Yaru Wang, Yitao Wang, Yu Wang, Wei Li, Ningkun Liu, Zhizhong Gong, Huilong Du

**Affiliations:** ^1^ College of Life Sciences, Institute of Life Science and Green Development Hebei University Baoding 071000 China; ^2^ Hebei Basic Science Center for Biotic Interaction Hebei University Baoding 071000 China; ^3^ State Key Laboratory of Plant Environmental Resilience, College of Biological Sciences China Agricultural University Beijing 100094 China

**Keywords:** allotetraploid, *Avena barbata*, B subgenome, genome evolution, subgenome differentiation

## Abstract

*Avena barbata*, a wild oat species within the genus *Avena*, is a widely used model for studying plant ecological adaptation due to its strong environmental adaptability and disease resistance, serving as a valuable genetic resource for oat improvement. Here, we phased the high‐quality chromosome‐level genome assembly of *A. barbata* (6.88 Gb, contig N50 = 53.74 Mb) into A (3.57 Gb with 47,687 genes) and B (3.31 Gb with 46,029 genes) subgenomes. Comparative genomics and phylogenomic analyses clarified the evolutionary relationships and trajectories of A, B, C and D subgenomes in *Avena*. We inferred that the A subgenome donor of *A. barbata* was *Avena hirtula*, while the B subgenome donor was probably an extinct diploid species closely related to *Avena wiestii*. Genome evolution analysis revealed the dynamic transposable element (TE) content and subgenome divergence, as well as extensive structure variations across A, B, C, and D subgenomes in *Avena*. Population genetic analysis of 211 *A. barbata* accessions from distinct ecotypes identified several candidate genes related to environmental adaptability and drought resistance. Our study provides a comprehensive genetic resource for exploring the genetic basis underlying the strong environmental adaptability of *A. barbata* and the molecular identification of important agronomic traits for oat breeding.

## Graphical abstract

A high‐quality genome sequence of the tetraploid wild oat *Avena barbata* provides crucial genetic information for the *Avena* B‐genome, allowing clarification of the evolutionary trajectories of the four subgenomes within *Avena* and identification of numerous key genes related to environmental adaptability, thus offering valuable resources for oat breeding and improvement.

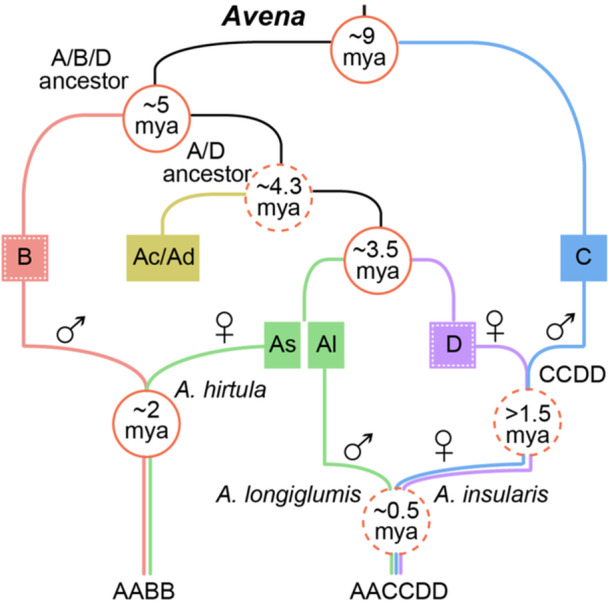

## INTRODUCTION

Common oat (*Avena sativa* L., 2*n* = 6*x* = 42, AACCDD), a member of the genus *Avena* within the Poaceae family (Soreng et al., 2017), is a globally significant cereal crop and is widely recognized as a healthy and nutritious whole‐grain food (Rasane et al., 2015). In addition, oats exhibit greater tolerance to adverse conditions compared with wheat, rice, and other crops and serve as a significant resource of high‐quality forage for livestock worldwide due to their rich fiber, protein (11%–15%), minerals, and a well balanced amino acid content (Ahmad et al., 2014). The genus *Avena* comprises approximately 30 species, including several edible species and invasive weeds, and encompass three natural ploidy levels: diploid, tetraploid, and hexaploid (Yan et al., 2016a; Liu, 2017). Most wild oats historically originated from the ancient Mediterranean region and, as suggested by many researchers, emerged through multiple hybridization events (Loskutov et al., 2023; Yacer et al., 2024). Wild oat species are considered an extremely valuable and diverse gene pool for improving cultivated oat varieties.

Four cytogenetically distinct types of (sub)genomes, designated A, B, C, and D, are recognized in the genus *Avena* (Gnutikov et al., 2023). Diploid species possess either an AA or CC genome, while tetraploid species have an AABB or CCDD genome (Yan et al., 2016a), and hexaploid species have an AACCDD genome (Yan et al., 2016b). Nevertheless, the B and D genomes are known only from polyploid species, as no extant diploid species with these genomes have been identified. Evolutionary studies suggest that the A and C genomes, two distinct evolutionary lineages of *Avena* chromosome sets, probably diverged from a common ancestor from 5 to 25 million years ago (Mya) (Peng et al., 2018; Gnutikov et al., 2022). During its evolution, the A genome also gave rise to two variants: the B and D genomes (Chen and Armstrong, 1994; Linares et al., 1996). Recent studies have documented that common oat was thought to have originated approximately 0.5 Mya from the hybridization between an Al/As genome diploid ancestor and a CD genome tetraploid closely related to *Avena insularis*, which originated from an allotetraploidy event between a C genome and a D genome diploid (Peng et al., 2022). Species with AABB genomes, such as *Avena barbata*, *Avena vaviloviana*, and *Avena abyssinica* (Fu, 2018), share a close phylogenetic relationship with *Avena wiestii* or *Avena hirtula* (Peng et al., 2018). Multiple lines of evidence suggest that the A genome progenitor of these AB genome tetraploids is likely a diploid species with the As genome (Allard et al., 1994; Katsiotis et al., 1997; Peng et al., 2018). However, the progenitor of the B genomes in these species remains unknown and controversial. Moreover, the lack of comprehensive genome‐scale evidence has left the origins and phylogenetic relationships among the B, A, C, and D genomic lineages unresolved.

*Avena barbata* is a tetraploid species with the AB genome, characterized as a slender wild oat native to the Mediterranean basin (Irigoyen et al., 2001). It has been studied extensively in ecological research and serves as a model for understanding plant adaptation and acclimation to diverse environmental conditions (Latta et al., 2022). Additionally, *A. barbata* offers significant promise for plant breeding due to its recognized resistance to cereal cyst nematode, crown rust, and powdery mildew, making it a valuable resource in the development of disease‐resistant crops (Carson, 2009, 2010). The current limited understanding of the position and distribution of these resistance genes on the chromosomes of *A. barbata*, as well as their evolution during polyploidization, hinders the effective use of these wild oat germplasm in oat breeding. Besides, the substantial genome size of *Avena*, along with its highly repetitive sequences and high ploidy level, results in its genomic research lagging significantly behind that of other crops. Although reference genomes for hexaploid oats (AACCDD) (Kamal et al., 2022; Peng et al., 2022), tetraploid oats (CCDD) (Kamal et al., 2022; Peng et al., 2022), and diploid oats with AA (Maughan et al., 2019; Kamal et al., 2022; Peng et al., 2022) or CC (Maughan et al., 2019) genomes are now available, a high‐quality whole‐genome sequence for the B genome is still lacking, which is crucial for fully exploiting the genetic potential of *A. barbata* and its wild relatives.

In this study, we shed light on the evolutionary trajectory of the B genomes across the *Avena* lineages, and confirmed that the donor of the A subgenome in *A. barbata* was *A. hirtula*, while the donor of B subgenome was most probably an extinct diploid species closely related to the extant *A. wiestii*. By integrating comparative genomics and transcriptomics, we conducted thorough analyses of subgenome evolution, divergence, expression dominance, and candidate resistance gene identification. Population genetic analysis of 211 *A. barbata* accessions from distinct ecotypes helped us identify several candidate genes related to environmental adaptability and drought resistance. This genetic resource is instrumental in enhancing our understanding of *Avena* evolution and will aid in future molecular breeding initiatives aimed at improving oat varieties.

## RESULTS

### High‐quality genome and annotation of *A. barbata*

**Figure 1:**
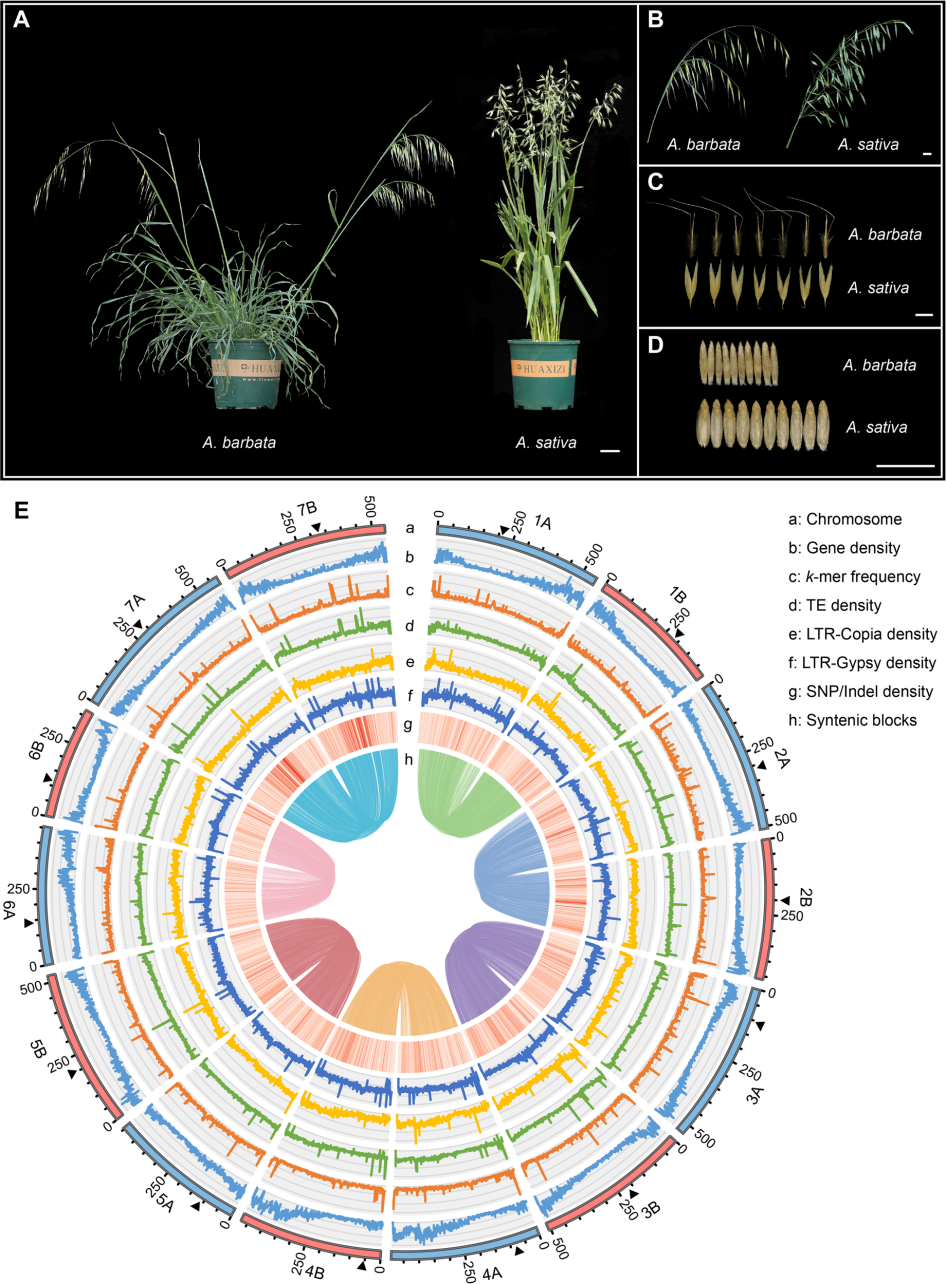
**Phenotype, assembly pipeline, and genomic landscape of** ***Avena barbata*** **(A–D)** Morphological features of *A. barbata* and *Avena sativa*, including the plant, spike type and seeds. Scale bars represent 2 cm. **(E)** Circos plot of the genomic features of the assembled *A. barbata* genome. From outer to inner ring: track “a–h” represents assembled chromosomes, gene density, *k*‐mer frequency, transposable element (TE) density, long terminal repeat (LTR)‐*Copia* density, LTR‐*Gypsy* density, single nucleotide polymorphisms (SNPs)/indels density, and pairwise syntenic blocks, respectively. The black triangle represents the location of the predicted centromere.

The genome size of *A. barbata* was estimated to be approximately 6.60 Gb via *k*‐mer (*k* = 21) analysis using Illumina sequencing data and 6.96 Gb via flow cytometry (Table S1; Figure S1). We assembled a 6.88 Gb genome of *A. barbata* using PacBio HiFi long‐read data and Hi‐C short read data (Tables 1, S1; Figure S2), which was consistent with the estimated genome size according to *k*‐mer analyses and flow cytometry. The assembled genome achieved a contig N50 of 53.74 Mb and consisted of 14 chromosomes (Table 1). Within the 14 chromosomes, we detected 18 telomeres, including six telomere‐to‐telomere chromosomes (Table S2). Benchmarking Universal Single‐Copy Orthologs (BUSCO) analysis indicated 97.80% genome completeness, and the long terminal repeat (LTR) Assembly Index (LAI) score was 15.63 (Table 1; Figure S2). Furthermore, 99.89% of the Illumina short reads were successfully aligned to the assembled genome. The consensus quality value (QV) of the genome was 71.95, and the assembly completeness was 97.44% based on HiFi reads (Table 1). Collectively, these results demonstrate the high continuity and completeness of the *A. barbata* genome assembly (Figure 1E).

**Table 1:** Statistics of *Avena barbata* genome assembly and annotation

	*Avena barbata*	A subgenome	B subgenome
Chromosome size (bp)	6,882,435,331	3,567,718,301	3,314,717,030
Contig numbers (bp)	283	162	103
Contig N50 (bp)	53,737,252	56,657,933	50,583,326
Scaffold N50 (bp)	507,161,011	508,443,857	473,029,490
Telomere	18/28	8/14	10/14
BUSCO	97.80%	96.40%	97.50%
Quality value (QV)	71.95	‐	‐
LAI	14.53	12.91	16.14
Repeats (%)	87.80	87.25	88.17
Protein‐coding genes (bp)	93,821	47,687	46,029
Average gene length (bp)	1,056	1,053	1,059

Approximately 6.05 Gb of the *A. barbata* genome was annotated as repetitive elements, accounting for 87.80% of the assembled genome (Table S3). Long terminal repeat retrotransposons (LTR‐RTs) comprised the major portion of transposable elements (TEs), representing 81.06% of the *A. barbata* genome (*Gypsy*: 42.81%, *Copia*: 18.34%, and Unknown: 19.91%) (Table S3). Furthermore, based on *ab initio* and evidence‐based predictions, in total 93,821 protein‐coding genes were annotated, with an average gene length of 2,901 bp, exon length of 264 bp, and average exon count of 4.0 (Table S4). Among these genes, 70.25% (65,910) were functionally annotated using four databases: InterProScan, Pfam, GO, and KEGG (Table S5).

The genome sequencing, assembly and annotation of *A. barbata* have been reported in a separate submission (Zhang et al., unpubl. data, 2025) and were used to construct the super pan‐genome of *Avena*.

### Subgenome phasing and centromere characterization

**Figure 2:**
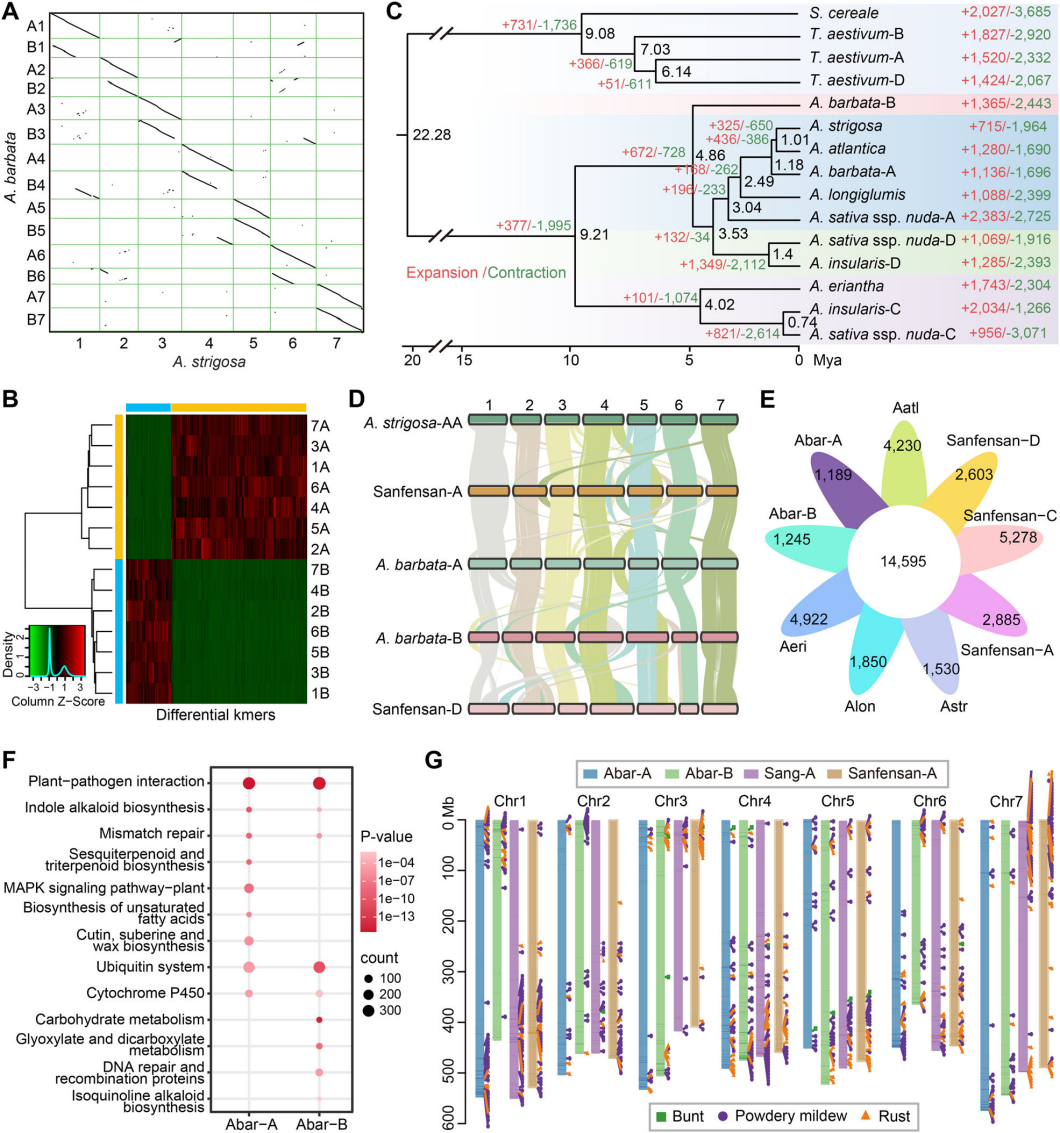
**The phylogenetic and gene family analysis for** ***Avena barbata*** **(A)** Genome‐wide synteny analysis between *Avena strigosa* and *A. barbata* using JCVI. **(B)** Unsupervised hierarchical clustering of *k*‐mers successfully phased *A. barbata* genome into two distinct subgenomes. **(C)** Phylogenetic relationships of 15 (sub)genomes from nine species based on single‐copy orthologs. Numbers in black represent the divergence time of each node (Mya, million years ago). The number of expanded and contracted gene families are shown in green and red, respectively. **(D)** The collinearity relationship among *A. strigosa*, Sanfensan, and *A. barbata*. **(E)** Venn diagram showing the numbers of common and unique gene families identified in nine oat (sub)genomes. Aatl: *Avena atlantica*; Alon: *Avena longiglumis*; Astr: *Avena strigosa*; Aeri: *Avena eriantha*; Abar: *Avena barbata*. **(F)** KEGG enrichment analysis for specific and expanded gene families in the A and B subgenome of *A. barbata*. **(G)** Genomic distribution of the R‐genes and the location of known resistance genes for powdery mildew, rust, and smut.

Two subgenomes of *A. barbata* were phased by combining the evidenced of phylogeny, specific *k*‐mers, and collinearity analysis. First, we employed SubPhaser to identify subgenome‐specific *k*‐mers, thereby segregating the 14 chromosomes into two distinct groups (Figure 2B). Furthermore, through synteny analysis with diploid *Avena strigosa* (AA), we designated the chromosomes in one group that showed closer relationships to *A. strigosa* as the A subgenome (chromosomes A1–7), while the remaining chromosomes in the other group were categorized as the B subgenome (chromosomes B1–7) (Figures 2A, S3). Additionally, we further confirmed the allotetraploid nature of *A. barbata* by assessing average nucleotide identity values, synonymous substitution rates (*K*_*s*_), and collinear relationships between its two subgenomes (Figure S3). Finally, the genome sizes of A and B subgenomes were 3.57 Gb with 47,687 protein‐coding genes and 3.31 Gb with 46,029 protein‐coding genes, respectively (Table 1).

Furthermore, we identified the centromere regions of *A. barbata* using previously reported chromatin immunoprecipitation sequencing (ChIP‐seq) data from cultivated oats (Liu et al., 2023), LTR distribution patterns, and *k*‐mer frequency analyses (Figure S4). The core centromeric regions spanned 4.9‐7.2 Mb (mean: 5.9 Mb) and 5.3‐8.1 Mb (mean: 6.3 Mb) in the A and B subgenomes, respectively (Table S6). Transposon elements accounted for 94.51% and 96.36% of centromeric sequences in the A and B subgenomes, predominantly *Gypsy*‐type retrotransposons. We identified 5,580 and 7,119 intact LTR‐RTs within the centromere regions of the A and B subgenomes, respectively, but no centromeric repeat units were detected in either subgenome. The centromeric regions of the A and B subgenomes exhibited significant differences in the proportion of distinct LTR‐RTs types, with particularly high abundances of CRM retrotransposons (Figure S5). Additionally, the LTR‐RTs in the centromeres showed recent insertion events, with an average age of less than 0.5 Mya, and distinct insertion time patterns were observed between the A and B subgenomes (Figure S5). Clustering analysis revealed that CRM retrotransposons form distinct groups consistent with subgenome classification (Figure S5). These findings revealed substantial sequence divergence within the centromeric regions among the subgenomes of *A. barbata*, reflecting its allopolyploid nature.

### Evolutionary relationship of A, B, C, and D subgenomes in *Avena*

To explore the evolutionary history of *A. barbata*, we conducted a gene family analysis using 15 (sub)genomes from nine cereal crop species including *Secale cereale*, *Triticum aestivum*, *A. strigosa*, *A. atlantica*, *A. longiglumis*, *A. sativa*, *A. eriantha*, *A. insularis*, and *A. barbata*. We constructed the phylogenetic tree and estimated species divergence times based on 6,408 single‐copy gene families identified among these species (Figure 2C). Species divergence times were estimated using MCMCtree in the *PAML* package (Yang, 2007). The established divergence times of *S. cereale*–*T. aestivum* (4.0–11.7 Mya) and *S. cereale*–*A. sativa* (22.4–31.8 Mya) were used for fossil calibration (http://www.timetree.org/). The results illustrated that the divergence between Aveneae and Triticeae was estimated to occur at approximately 22.28 Mya, and the C subgenome first diverged from the common ancestor of *Avena* species around 9.21 Mya (Figure 2C). Notably, the B subgenome of *A. barbata* diverged from the common ancestor of the A/D subgenomes at approximately 4.86 Mya (Figure 2C). Additionally, the divergence time between the A subgenome of hexaploid oats and *A. longiglumis* was 3.04 Mya, between D subgenome of hexaploid oats and that of *A. insularis* was 1.40 Mya, while between C subgenome of hexaploid oats and that of *A. insularis* was 0.74 Mya (Figure 2C), consistent with previous results (Peng et al., 2022). Furthermore, the *Ks* distribution for homologous genes between the A and B subgenomes was calculated to estimate the timing of the AABB genome polyploidization event. The peak *K*_*s*_ value of 0.0272 indicates that the AABB genome polyploidization occurred at approximately 2.0 Mya, calculated using the formula T = *K*_*s*_/(2 *μ*) with *μ* = 6.5 × 10^−9^ (Figure S3). In addition, pairwise synteny analysis revealed strong macrosynteny among the A and B subgenomes of *A. barbata* and the corresponding diploid AA genome of *A. strigosa*, as well as with the A and D subgenomes of hexaploids oats (Sanfensan) (Figure 2D). However, large‐scale genomic rearrangements were also observed, indicating that significant changes occurred following the polyploidization event (Figure 2D).

Next, we identified 14,595 gene families shared among nine subgenomes from six *Avena* species, along with 1,189 and 1,245 gene families specific to the A and B subgenomes of *A. barbata*, respectively (Figure 2E). In addition, 1,136 and 1,365 significantly expanded gene families were identified in the A and B subgenomes of *A. barbata*, respectively (Figure 2C). KEGG enrichment analysis revealed that these specific and expanded gene families in the A and B subgenomes are mainly associated with environmental adaptabilities, such as “plant–pathogen interaction,” “indole alkaloid biosynthesis,” and “cytochrome P450” (Figure 2F), consistent with *A. barbata*'s strong resistance to a variety of diseases (Carson, 2009). We further identified 442, 408, 423, 301, 417, 448, 343, and 449 resistance genes (R‐genes) in the *A. barbata*‐A, *A. barbata*‐B, Sanfensan‐A, Sanfensan‐C, Sanfensan‐D, Sang‐A, Sang‐C, and Sang‐D subgenomes, respectively (Figure S6). Although the numbers of R‐genes in the A and B subgenomes of *A. barbata* were similar to those in the hexaploid subgenomes, except for the C subgenome, many specific and expanded R‐gene families and loci were identified (Figure 2G). Furthermore, based on previously cloned resistance genes against powdery mildew, rust, and black smut in Poaceae species, we detected 155, 149, 212, 163, 207, 225, 178, and 231 homologous genes in the *A. barbata*‐A, *A. barbata*‐B, Sanfensan‐A, Sanfensan‐C, Sanfensan‐D, Sang‐A, Sang‐C, and Sang‐D subgenomes, respectively (Figure S6). Our analysis revealed that most of these disease‐resistant genes were clustered at the distal ends of the chromosome arms across all subgenomes (Figure 2G). Notably, significant expansions of disease‐resistant genes were observed in the chromosomal heads of 1A, 1B, 2A, and 2B in *A. barbata* compared with cultivated oats, showing promising candidates loci for the improvement of oat disease resistance (Figure 2G).

### Potential ancestral species of A and B subgenomes in *A. barbata*

**Figure 3:**
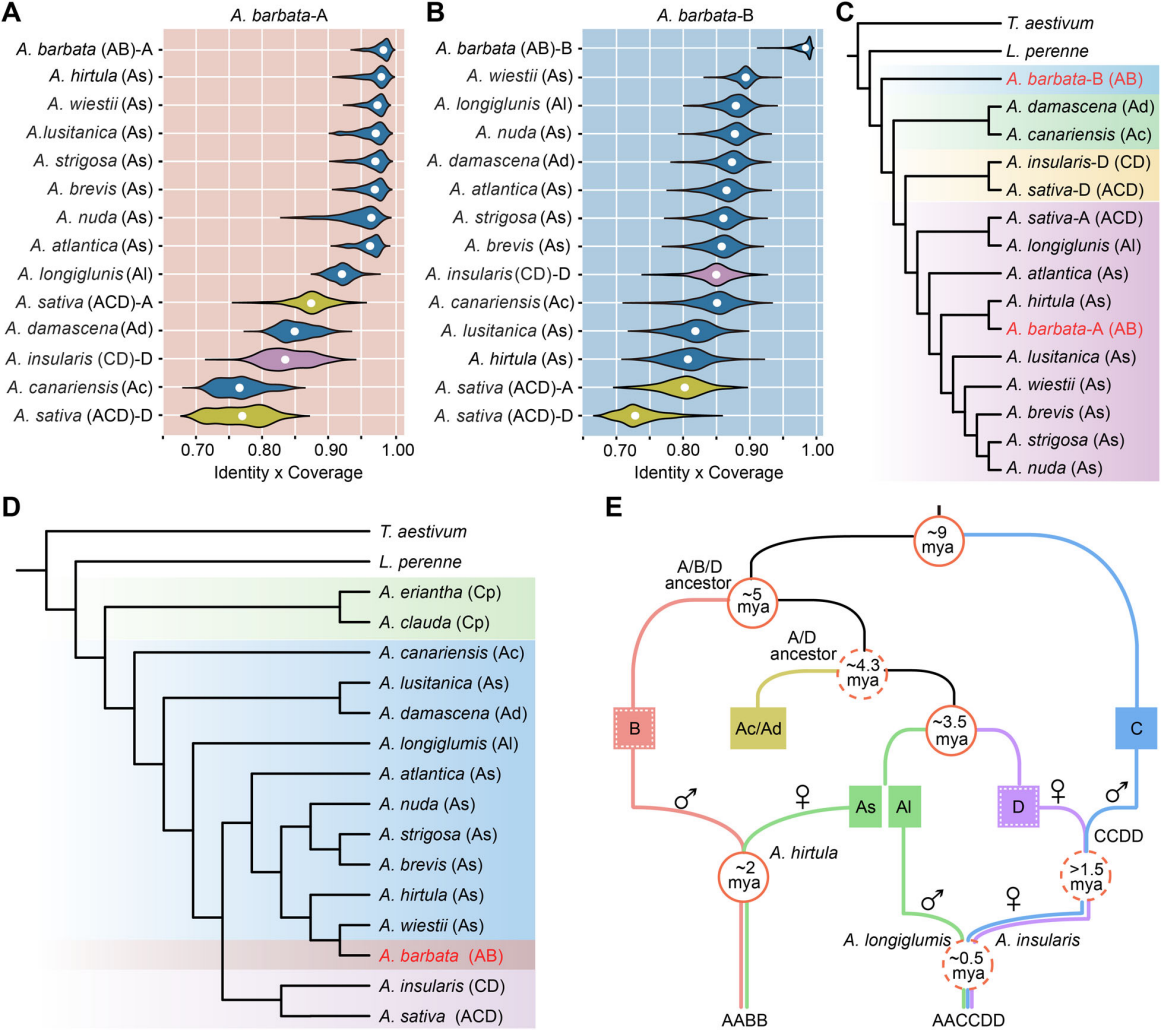
**Phylogenetics and polyploidization of** ***Avena*** **species** **(A, B**) Sequence similarities of reads from various *Avena* species that were uniquely mapped to the A and B subgenomes of *Avena barbata*. **(C)** Phylogenetics of various (sub)genomes generated from the single nucleotide polymorphisms (SNPs) using Sanfensan‐A as a reference. **(D)** Unrooted phylogenetic tree constructed using the complete sequences of the *Avena* chloroplast genomes. **(E)** Model showing the evolutionary history of tetraploid oat (*A. barbata*; AABB) and hexaploid oat (*A. sativa*; AACCDD). The differentiation times within the solid circle are derived from this study, whereas the time within the dashed circle relies on prior findings (Peng et al., 2022).

Previous phylogenetic studies struggled to differentiate between the A and B subgenomes, leading to confusion and inconsistent findings regarding the origins of the AABB genomes (Gnutikov et al., 2023). Furthermore, phylogenies based solely on chloroplast and mitochondrial genomes limited the exploration of evolutionary relationships for paternal subgenomes for polyploid species. To accurately identify the subgenome donors, we downloaded the resequencing data of 12 *Avena* species representing different genomic subtypes and ploidy levels (As, Al, Ac, Ad, CD, and ACD) based on the genome assemblies of the A and B subgenomes (Table S7). All resequencing data were mapped to the A and B subgenomes. Identity and coverage analysis revealed that the diploid As species *A. hirtula* exhibited the highest value of similarity times coverage (approximately 97.9%) to the A subgenome of *A. barbata* (Figure 3A). In contrast, although the diploid As species *A. wiestii* exhibited the highest value of similarity times coverage (89.7%) to the B subgenome of *A. barbata*, this value was significantly lower than that observed between *A. hirtula* and the A subgenome of *A. barbata* (Figure 3B). Furthermore, a single nucleotide polymorphism (SNP)‐based phylogenetic tree constructed using the A genome of Sanfensan as a reference indicated that *A. hirtula* was most closely related to the A subgenome of *A. barbata*, while none of the extant diploids showed a particularly close relationship with B subgenome of *A. barbata* (Figure 3C). These findings suggested that *A. hirtula* was A genome progenitor of *A. barbata* (Allard et al., 1994), while the donor of the B subgenome was most probably an extinct diploid species closely related to the extant species *A. wiestii*.

Subsequently, we assembled the chloroplast genomes of *A. barbata* and constructed a phylogenetic tree in combination with the 16 previously reported chloroplast genomes to determine the maternal genome donor of *A. barbata* (Figure 3D; Table S8). Our phylogenetic analysis based on the chloroplast tree showed that the As genome was the maternal donor in *A. barbata*, while our phylogenetic analysis based on the subgenome species tree showed the earliest divergence of the B subgenome of *A. barbata* from the common ancestor of the A/D subgenomes (Figure 3D). These results indicated that the B genome was undoubtedly the male parent in the polyploidization event, while the A genome was the maternal donor in *A. barbata*. Further combining with the previous report on the evolutionary history of the A, C, and D subgenomes in hexaploid oats (Peng et al., 2022), we proposed a model for the origins, polyploidizations, and evolutionary trajectories of all subgenomes (A, B, C, D) in *Avena* (Figure 3E). The C and A/B/D lineages diverged approximately 9 Mya, followed by the divergence of the B subgenome from the common ancestor of the A and D lineages around 5 Mya, and then the A genome subtypes (Ac/Ad) and the D genome around 3.5 Mya (Figure 3E). The AB genome in *A. barbata* is estimated to have originated approximately 2.0 Mya through hybridization between a maternal As genome diploid from *A. hirtula* and a paternal B genome closely related to *A. wiestii* (Figures 3E, S3). These findings clarified the evolutionary trajectories of tetraploid oats (AABB and CCDD) and hexaploid oats (AACCDD) based on various pieces of genomic evidence and offer key clues to the subgenomic origins within *Avena*.

### Genome evolution and divergence of A, B, C, and D subgenomes in *Avena*

**Figure 4:**
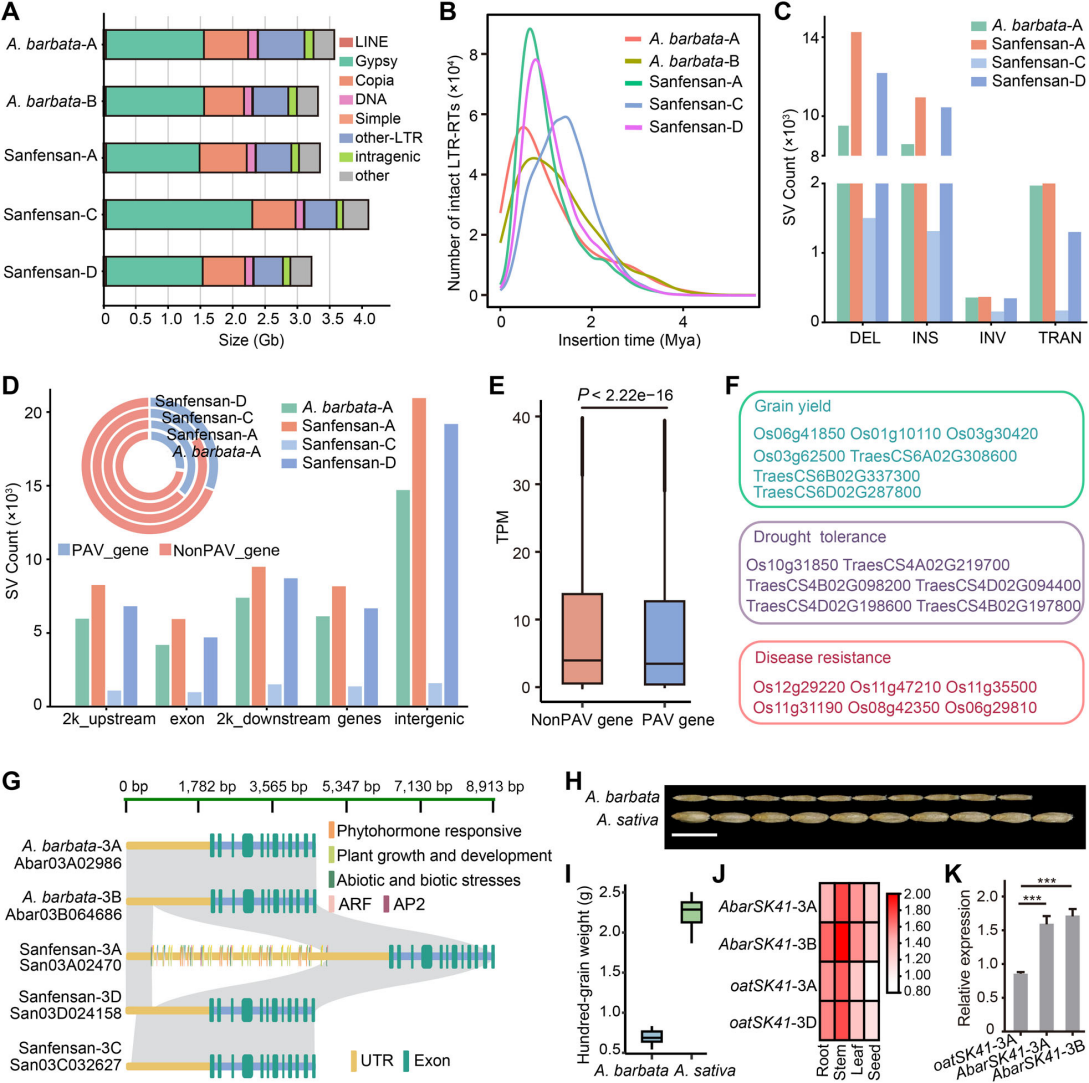
Transposable element (TE) content and structural variation across different oat subgenomes **(A)** Phylogenetic tree and genomic constituents of five subgenomes of *Avena barbata* and cultivated oats Sanfensan. **(B)** Temporal patterns of long terminal repeat retrotransposons (LTR‐RTs) insertional bursts in subgenomes of *A. barbata* and Sanfensan. **(C)** Structural variation (SV) number and type in *A. barbata*‐A, Sanfensan‐A, Sanfensan‐C, and Sanfensan‐D subgenome using *A. barbata*‐B subgenome as reference **(D)** Numbers of SVs overlapping with different genomic features in each subgenome. The pie chart represents the SV number and type in four subgenomes. **(E)** Expression levels of SV‐associated and non‐SV genes (two‐tailed Student's *t*‐test, ***P < 0.001). **(F)** Functional classification of the agronomically important genes associated with B subgenome‐specific SV. **(G)** Gene structures of homologous *OsSK41* genes in *A. barbata* and Sanfensan. **(H)** Morphological features of seeds in *A. barbata* and Sanfensan. **(I)** Comparison of hundred grain weight in *A. barbata* and Sanfensan. **(J**, **K)** The seed expression of homologous *OsSK41* genes in *A. barbata* and Sanfensan with transcriptome and quantitative real‐time polymerase chain reaction (qRT‐PCR).

Understanding the subgenome repeat content and structural variations (SVs) is essential for unraveling the genomic organization and evolutionary dynamics of polyploid species. We compared the TE content across different subgenomes. The TE content in the *A. barbata*‐B subgenome (2.89 Gb) was 87.25%, slightly higher than that in the Sanfensan‐D subgenome (87.15%, 2.81 Gb), but lower than that in the *A. barbata*‐A (88.17%, 3.15 Gb), Sanfensan‐A (87.69%, 2.94 Gb), and Sanfensan‐C (88.64%, 3.62 Gb) (Figure 4A). Notably, the C subgenome (3.62 Gb) had the highest proportion of repetitive sequences, with approximately 0.68, 0.73, and 0.81 Gb more repetitive sequences than A, B, and D subgenomes, respectively, which may be a major contributing factor to its subgenome size expansion (Figure 4A). The LTR‐*Gypsy* type was the most abundant, constituting 45.85%, 42.44%, 43.17%, 46.46%, and 43.75% of *A. barbata*‐A, *A. barbata*‐B, Sanfensan‐A, Sanfensan‐C, and Sanfensan‐D subgenomes, respectively (Figure 4A). We identified a total of 134,736, 119,733, 98,660, 127,055, and 93,645 intact LTR‐RTs in *A. barbata*‐A, *A. barbata*‐B, Sanfensan‐A, Sanfensan‐C, and Sanfensan‐D, respectively (Table S9). Using the molecular clock hypothesis (SanMiguel et al., 1998; Kijima and Innan, 2010), the insertion times of intact LTR‐RTs into the genome were estimated by examining the divergence between the 5′ and 3′ long terminal repeat sequences. The results revealed peaks at 0.45, 0.73, 0.61, 1.45, and 0.81 Mya for the insertions in *A. barbata*‐A, *A. barbata*‐B, Sanfensan‐A, Sanfensan‐C, and Sanfensan‐D, respectively, indicating variability in LTR‐RT insertion times across subgenomes (Figure 4B). Meanwhile, the *A. barbata*‐B subgenome contained the highest number of *Gypsy*‐CRM subclasses, totaling 8,438, which was more than that found in the other subgenomes, ranging from 3,811 to 7,227 (Table S9). The Sanfensan‐C subgenome had the lowest number, with only 3,811 *Gypsy*‐CRM subclasses (Table S9). The identification and comparison of TE families between cultivated and wild oats or among subgenomes could enhance our understanding of the dynamic changes in TEs and their impacts on genome evolution in *Avena*.

Taking the *A. barbata*‐B subgenome as the reference, in total 19,668, 29,008, 3,142, and 24,392 SVs (>50 bp) comprising 7,764, 11,008, 1,314, and 10,503 insertions, 9,577, 14,316, 1,501, and 12,242 deletions, 1,969, 3,317, 170, and 1,301 translocations (TRANS) and 358, 367, 157, and 346 inversions (INVs), were identified in *A. barbata*‐A, Sanfensan‐A, Sanfensan‐C, and Sanfensan‐D, respectively (Figures 4C, S7; Table S10). Approximately 90% of these insertions, deletions, INVs, and TRANS were shorter than 110, 200, 20, and 450 kb, respectively (Figure S8; Table S11). To confirm the precision of the identified SVs, we randomly sampled 50 SVs and verified them using HiFi read mapping and Hi‐C contact map analysis: only two boundaries deviated from the mapped results, suggesting high reliability in SV detection (Figures S9, S10). Among the five subgenomes, presence/absence variants (PAVs) were the most prevalent type of SV and were predominantly found in intergenic regions, with 89.67% overlapping with TEs (Figure 4D). These TE‐associated PAVs were particularly enriched in LTR retrotransposon regions, with *Gypsy* elements being the most prominent, indicating that the recent expansion of LTR‐RTs has significantly contributed to the generation of numerous PAVs in oats (Table S12). Furthermore, we identified 16,514 genes affected by PAVs, which generally exhibited reduced expression levels (Figures 4E, S11). GO enrichment analysis revealed that these PAV‐affected genes were predominantly involved in stress response, cellular responses to abscisic acid stimuli, and terpene synthase activity (Figure S11). Additionally, we discovered 730 PAVs unique to the B genome, affecting 201 genes, many of which are associated with crucial traits such as yield, drought resistance, and disease resistance (Figure 4F; Table S13).

Among these genes affected by PAVs, a notable example is the identification of a 4,298‐bp insertion located upstream of the *SHAGGY*‐*like kinase 41* (*SK41*) gene on chromosome 3A in the cultivated oat genome, which is absent in the homologous genes of *A. barbata* (Figure 4G). It is observed that *A. barbata* mostly displays smaller grains and lower weight compared with cultivated oats (Figure 4H, I). Transcriptome and quantitative real‐time polymerase chain reaction (qRT‐PCR) analyses demonstrated significantly lower expression levels of this gene in the seeds of the Sanfensan‐A subgenome compared with the other three subgenomes (Figure 4J, K). Furthermore, we also identified 54 putative regulatory elements within the inserted sequence, including promoters, *cis*‐regulatory elements, and 10 transcription factors (Figure 4G). These findings align with a prior study identifying *SK41* as a negative regulator involved in controlling grain size and weight in rice (Hu et al., 2018).

### Subgenome expression dominance in *A. barbata*

**Figure 5:**
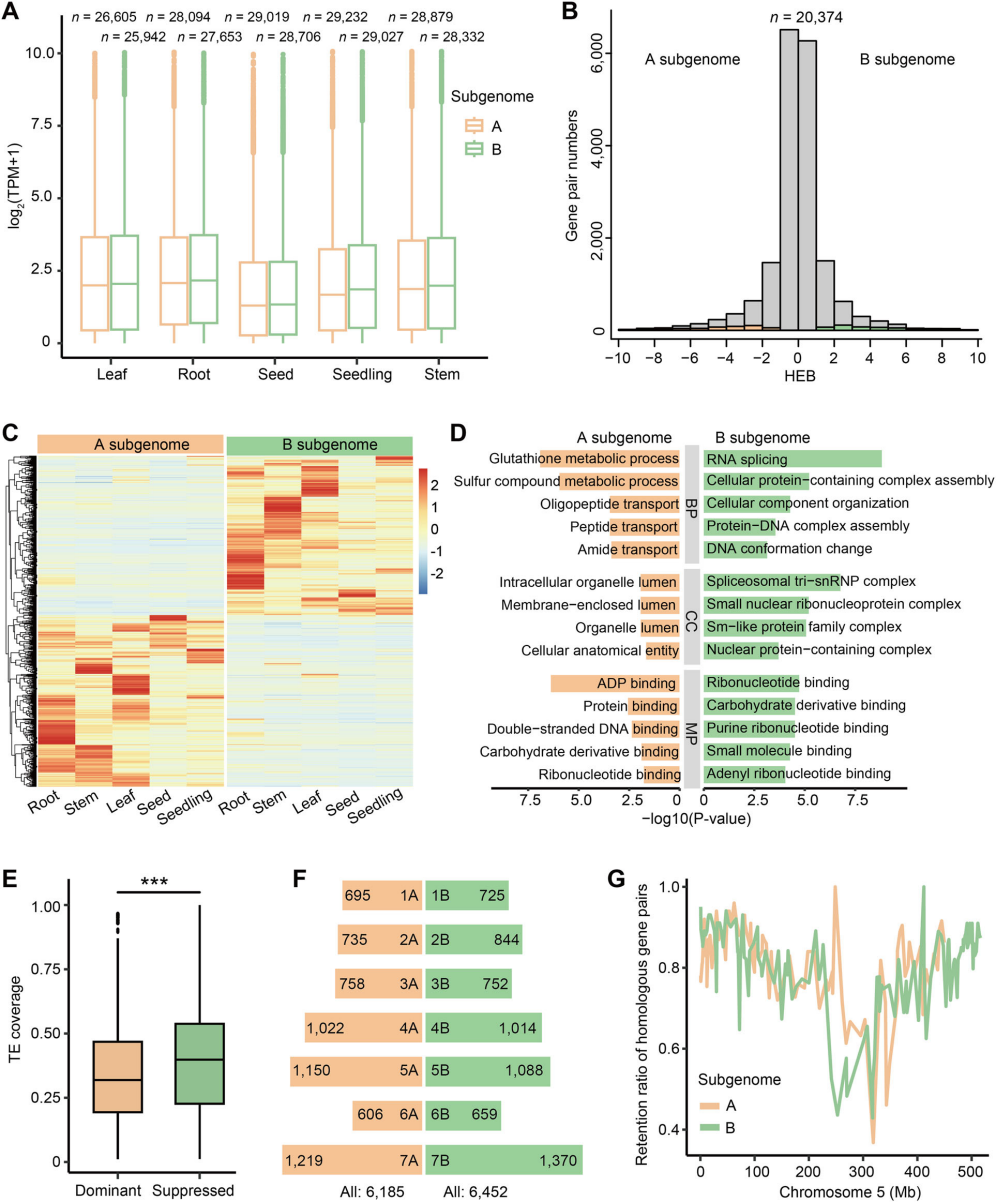
**Subgenome dominance in the** ***Avena barbata*** **genome** **(A)** Boxplot showing expression levels of all the expressed genes (FPKM ≥ 0.1) across different tissues in the A and B subgenomes of *A. barbata*. The numbers of expressed genes in the different tissues of subgenomes are given at the top. **(B)** Expression bias between homoeologs in the *A. barbata* genome. **(C)** Gene expression heatmap of gene pairs with significant HEB between A and B subgenomes. **(D)** Enriched Gene Ontology (GO) terms among homoeolog pairs that are significantly biased toward the A (orange) or B (green) subgenome. **(E)** Repetitive element coverage of gene regions with dominant and suppressed expression. **(F)** The number of predicted lost genes in the A (orange) or B (green) subgenome. **(G)** Plot of gene retention rates within synteny blocks across two subgenomes in chromosome 5.

In polyploid plants, quantitative variation for many agronomic traits is modulated by genetic interactions between multiple sets of homoeologs in different subgenomes (Alger and Edger, 2020). To investigate subgenome expression dominance in *A. barbata*, we conducted a comprehensive transcriptome analysis using 15 samples from five *A. barbata* tissues: root, stem, leaf, seed, and aboveground seedling parts. The number of genes expressed in the B subgenome (25,942–29,027) was comparable with that in the A subgenome (26,605–29,232) across all tissues (Figure 5A). Gene expression profiling revealed that the majority of expressed genes (88%–90%) were homoeologous between subgenomes, with only 10%–12% being subgenome specific. These findings suggest that there is no significant subgenome dominance in the gene expression patterns of *A. barbata*. Among the 20,374 homoeologous gene pairs within collinear blocks, we identified 2,214 gene pairs with a higher homoeolog expression bias (HEB; HEB > 2) toward one subgenome in all tissues (Figure 5B). Specifically, 1,133 genes were biased toward the A subgenome, and 1,081 toward the B subgenome (Figure 5C). Genes with higher HEB in the A subgenome were enriched in processes such as glutathione metabolism and sulfur compound metabolism, while those in the B subgenome were enriched in RNA splicing and ribonucleotide binding (Figure 5D). These results illustrate that the A and B subgenomes may contribute distinctively to specific environmental adaptation and phenotypic diversification in *A. barbata*.

We also investigated the average *Ka/Ks* ratios for genes showing higher (dominant) expression and lower (suppressed) expression in the A subgenome, as well as neutral expression (i.e., gene pairs without significant differences in expression between the A and B subgenomes) (Figure S12). The dominant and suppressed genes had significantly higher *Ka/Ks* ratios (median: 0.346 and 0.345, respectively) than the genes with neutral expression (median: 0.269) (Figure S12). Average *Ka* ratios also followed the same trend as *K*_*a*_ */K*_*s*_ ratios, indicating that dominant and suppressed genes evolved faster than neutral genes (Figure S12). Furthermore, we noted a significantly lower TE content near genes with dominant expression compared with those with suppressed expression, which supported the observed negative correlation between gene expression levels and nearby TE density reported in previous studies (Alger and Edger, 2020) (Figure 5E).

In addition, we examined the possible loss of genes during the evolution of the *A. barbata* genome. Within the conserved collinear regions, in total 12,637 genes were predictably lost, with 6,185 genes lost in the A subgenome and 6,452 genes lost in the B subgenome (Figure 5F). We noted that the majority of lost genes were primarily located in the pericentromeric regions of the chromosomes (Figures 5G, S13). The lost genes in the B subgenome were enriched in biological processes related to the regulation of gene expression, detection of external stimulus, and NADH dehydrogenase activity (Figure S14). In contrast, the lost genes in the A subgenome contained more members with essential biological functions, such as the macromolecule metabolic process, nitrogen compound metabolic process, and ubiquitin‐protein transferase regulator activity (Figure S14). Significantly, we detected a notably higher coverage of TEs within the gene loss regions, indicating that TEs likely play a critical role in both gene expression and the occurrence of gene loss. These results suggested that the different patterns of gene loss may have contributed to the distinct evolutionary trajectories of the two subgenomes and promoted the functional differentiation of the A and B subgenomes in *A. barbata*.

### Identification of adaptive‐related genes

**Figure 6:**
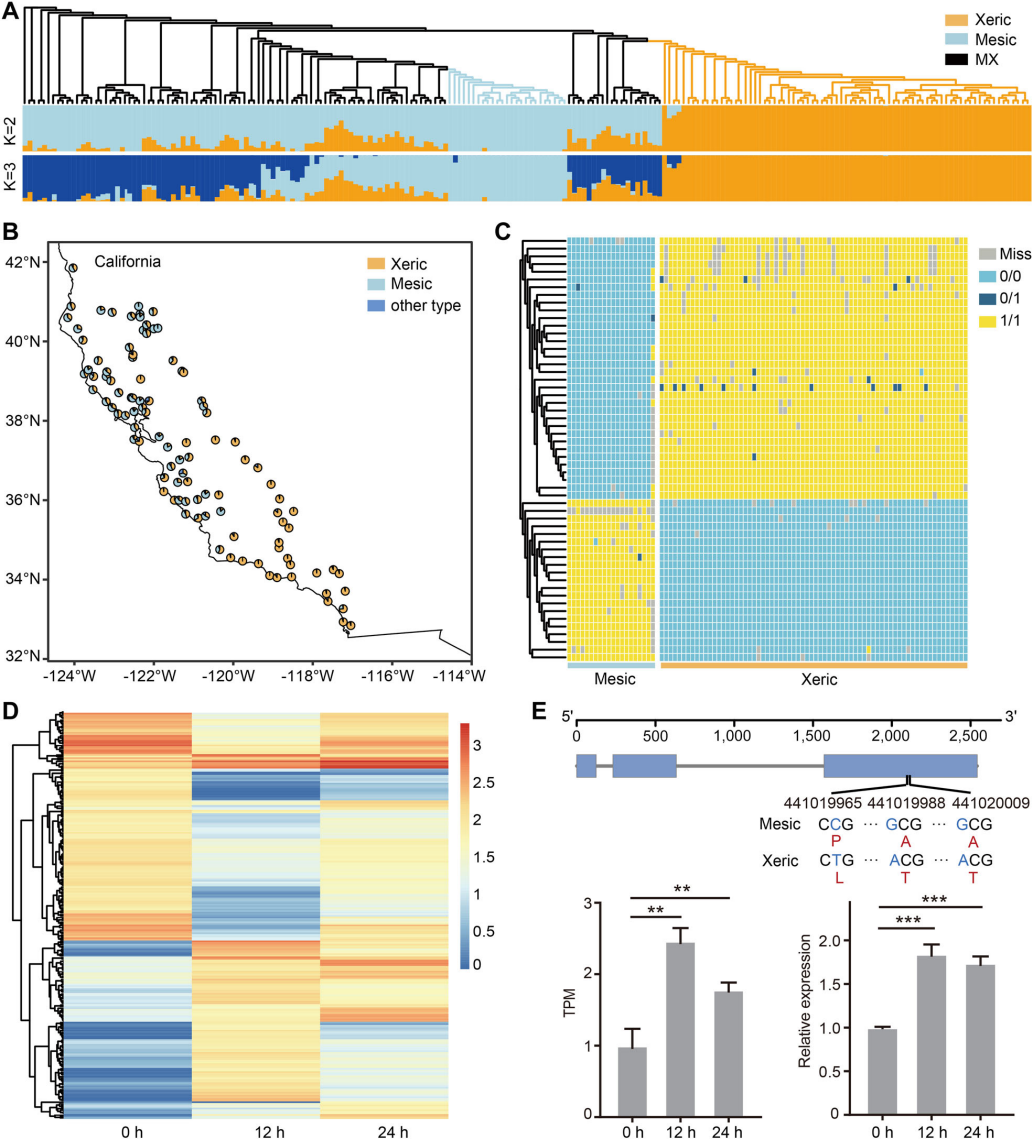
Identification of environmental adaptation‐related genes **(A)** Maximum likelihood phylogenetic tree and population structure of 211 *A. barbata* accessions based on GBS data. **(B)** The proportion of xeric (orange) and mesic (blue) ecotypes across 100 sites in California. **(C)** Single nucleotide polymorphisms (SNPs) present in 48 genes distinguish between xeric (orange) and mesic (blue) ecotypes. **(D)** Heatmap of differential expression genes following 0, 12, and 24 h of drought stress. **(E)** Gene structures and expression of homologous *AtIDD14* in *Avena barbata* (*Abar04A0128581*). Three nonsynonymous SNPs in coding regions of *Abar04A0128581* distinguished xeric and mesic populations.

The *A. barbata* populations in California have been the subject of extensive research as one of the most studied “ecotypes” (Latta et al., 2022). Early studies divided the *A. barbata* population into two ecotypes: xeric and mesic, postulating a strong correlation between these ecotypes and local rainfall and temperature conditions (Clegg and Allard, 1972). To delve into the adaptive evolutionary processes of *A. barbata* and pinpoint genes involved in environmental adaptation, we performed a population genomics analysis utilizing these publicly available genotyping by sequencing (GBS) data from 211*A. barbata* accessions from distinct ecotypes (Table S14). Our high‐quality *A. barbata* genome provides an unprecedented opportunity to obtain the variation landscape hidden in these resequencing data. These GBS data were aligned to the *A. barbata* genome, yielding a dataset of 102,863 high‐quality SNPs. The phylogenetic tree was constructed based on these SNPs, and the analysis of population structure revealed a clear distinction between the xeric and mesic subpopulations (Figure 6A). In addition to these two subpopulations, a subset was identified that exhibited mixed characteristics of these two subpopulations. This finding indicated the likelihood of hybridization and recombination events occurring between these two subpopulations. The *F*3‐statistics strongly support this hypothesis, with a significant *Z* score of −4.52536 (Table S15). This subset was thus designated as MX recombinants.

Given the strong correlation between the xeric and mesic ecotypes of *A. barbata* and environmental factors such as rainfall, we used these ecotypes to investigate genes linked to environmental adaptation (Figure 6B) (Latta et al., 2022). By analyzing SNPs from both xeric and mesic populations, we identified 55 key SNPs that distinctly differentiated between the two ecotypes, implicating 48 genes (Figure 6C). Among the genes identified, 16 were known to play crucial roles in responses to both biological and abiotic stressors (Table S16). Notably, nine of these genes exhibited differential expression under drought stress conditions in our transcriptome data following 0, 12, and 24 h of drought stress, and the expression of five genes was validated through qRT‐PCR (Figures 6D, S15–17). Additionally, we selected a candidate gene, *Abar04A0128581* (IDD14), which has been reported to interact with ABFs in *Arabidopsis thaliana*, and actively modulate the plant's response to drought stress (Liu et al., 2022). Three nonsynonymous SNPs were identified within coding regions of *Abar04A0128581*, which can be clearly distinguished between the xeric and mesic populations (Figure 6E). Transcriptome and qRT‐PCR results showed that the expression level of *Abar04A0128581* was significantly upregulated after drought treatments, suggesting that it may be one of the key genes related to *A. barbata* drought resistance (Figure 6E). These results provide an important resource that will facilitate the molecular identification of important agronomic traits and the studies of the genetic basis underlying the strong environmental adaptability of *A. barbata*.

## DISCUSSION

*A. barbata* is a tetraploid wild oat species with the AB genome, which encompasses the B subgenome and is recognized as a significant source of resistance to various oat diseases (Carson, 2009, 2010). In this study, we elucidate that *A. barbata* is an allotetraploid species, originating from two distinct diploid *Avena* species. We ascertained that the donor of the A subgenome was the diploid *A. hirtula* species, aligning with prior findings derived from GBS markers (Allard et al., 1994; Latta et al., 2019). Conversely, the B subgenome's progenitor was most likely an extinct diploid species closely related to the extant diploid *A. wiestii* species. Previous studies have proposed that the B subgenome diploid ancestor may have been a genetically homogeneous species that hybridized multiple times with a more prevalent and genetically heterogeneous A genome species (Soltis et al., 2004; Latta et al., 2019).

The release of the cultivated oat genome illuminates the evolutionary trajectories of the A, C, and D subgenomes during polyploidization (Peng et al., 2022). To date, little progress has been made in elucidating the origins and evolution of the B subgenome in *Avena*. Here, we shed light on the evolutionary trajectory of tetraploid oats (AABB) and hexaploid oats (AACCDD). We further deduced that the polyploidization event of the AABB genome occurred at approximately 2.0 Mya. Notably, it was generally accepted that the B/D genome diverged from the A genome due to the high homology between the B and A genomes, as well as between the D and A genomes (Chen and Armstrong, 1994; Linares et al., 1996; Katsiotis et al., 1997). However, based on various pieces of genomic evidence, we found that the B genome was the earliest to diverge from the common ancestor of the A/B/D lineages at approximately 5 Mya, consistent with previous speculations based on GBS markers analysis (Latta et al., 2019). This discovery marks that the evolutionary positions of all subgenomes and lineages in *Avena* have been elucidated.

Polyploidy serves as a significant catalyst for plant evolution and is prevalent in *Avena* (Alger and Edger, 2020; Tomaszewska et al., 2022). Analyses at the subgenome level have uncovered asymmetrical evolution of parental subgenomes in numerous allopolyploid species, and a HEB has also been observed, such as in octoploid strawberry (Edger et al., 2019) and *Brassica juncea* (Yang et al., 2016). The notion of subgenome dominance is firmly entrenched in allopolyploid species, however, certain allopolyploids, such as horseradish (Shen et al., 2023) and pumpkin (Sun et al., 2017), lack this feature. Interestingly, the two published cultivated oat genomes presented contradictory conclusions regarding the presence of subgenomic dominance: the Sanfensan genome showed subgenomic dominance (Peng et al., 2022), while the Sang genome exhibited a balanced expression among three subgenomes (Kamal et al., 2022). In the case of *A. barbata*, distinct subgenome dominance was not observed. However, we found that genes associated with different biological processes exhibited subgenome‐specific biases in gene expression. The notable differences in homoeologous gene expression were strongly correlated with distances to TEs, highlighting the pivotal role of TE insertions in molding the *A. barbata* genome and its biological characteristics. Similar observations have been made in the octoploid strawberry genome, where density variations of TEs proximate to homoeologs have been implicated in divergent gene expression (Edger et al., 2019). Our findings here revealed a significantly elevated coverage of TEs near genes with suppressed expression and within regions of gene loss. Consequently, we hypothesize that the variance in TE insertion abundance, particularly for LTR‐RTs, contributes to the observed subgenome dominance (Shen et al., 2023).

*A. barbata* has been shown to contain many beneficial traits, particularly in terms of resistance to various diseases and tolerance to drought, cold temperatures, and poor soil fertility (Carson, 2009, 2010). These characteristics render the species a crucial genetic resource for potential oat improvement. Over the past few decades, interspecific crosses within the Avenae tribe have been effectively utilized to transfer these disease‐resistant traits into cultivated oats, primarily for improving disease resistance (Thomas et al., 1975). Leveraging the high‐quality genome generated from this study, we have exhaustively identified R‐genes and homologs of previously cloned resistance genes against powdery mildew, rust, and black smut across different subgenomes. Notably, we found several loci with significant expansions of disease‐resistant genes in *A. barbata* compared with cultivated oats, presenting potential resources for improving disease resistance. Moreover, *A. barbata* was extensively studied in California using allozymes in the 1970s and was interpreted as a case of ecotypic adaptation to contrasting moisture environments (Allard et al., 1972). The original distinction between mesic and xeric ecotypes was based on the observation of two multilocus allozyme genotypes (Allard et al., 1972; Latta et al., 2022). The assembly of the *A. barbata* genome is poised to uncover variations that were previously undetectable with a limited number of allozyme loci, benefiting research on the adaptive evolution of *A. barbata* in California. Based on these data, we have also identified a plethora of genes associated with environmental adaptation, offering a vital reference for oat improvement.

In summary, this study contributes to our understanding of the origins of the A/B subgenomes and the evolution of polyploid genomes, but also helps to clarify the evolutionary history of the four subgenomes within *Avena*, laying a stable foundation for further mining of valuable genes and novel alleles for oat improvement.

## MATERIALS AND METHODS

### Plant materials, growth conditions, and treatments

*Avena barbata* Pott. ex Link. (2*n* = 4*x* = 28, AABB) was sourced from the National Germplasm Bank of China (NGBC). The plants were grown in the greenhouse at 25°C/18°C under a 12‐h light/12‐h dark photoperiod. When the *A. barbata* seedlings reached the trifoliate stage, they were exposed to 20% polyethylene glycol (PEG) 6000 to simulate drought conditions. The aerial parts of the plants were collected at specific time intervals (0, 12, and 24 h) following the initiation of PEG6000 treatment. All samples were collected at the same time of day and immediately frozen in liquid nitrogen for RNA extraction, with three biological replicates for each tissue type.

### Genome assembly and annotation of *A. barbata*

The genome sequencing, assembly and annotation information of *A. barbata* has been reported in a separate submission (Zhang et al., under review in *Nature Genetics*), and the final assembly of *A. barbata* was mainly used to construct the super pan‐genome of *Avena*.

The genome size was estimated by flow cytometry using wheat as an internal control, following the previously described method (He et al., 2024). The *k*‐mer distribution frequency was calculated using Jellyfish (v2.3.0) (Marcais and Kingsford, 2011) with the Illumina and HiFi reads, and estimated the genome characteristics using GenomeScope (v2.0) (Ranallo‐Benavidez et al., 2020). To assess the integrity of the assembly and the consistency of the sequencing data, Illumina reads were aligned to the final assembly using BWA (v0.7.17) (Vaser et al., 2017). BUSCO (v5.2.2) (Manni et al., 2021) was used to evaluate the completeness of gene structures, employing the embryophyta_odb10 dataset as a reference. LTR_retriever (v2.9.0) (Ou and Jiang, 2018) was applied to identify LTR retrotransposons, while the LAI was computed to measure the completeness of repeat‐rich regions. Ultimately, the precision of the assembly was estimated using Merqury (v1.1) (Rhie et al., 2020) software with HiFi reads.

Repetitive sequences in the *A. barbata* genome were annotated using both *de novo* prediction and homology‐based methods. RepeatModeler (v1.0.11) (Robert Hubley, 2010), LTR_FINDER (v1.07) (Xu and Wang, 2007), LTRharvest (Ellinghaus et al., 2008) and LTR_retriever (v2.9.0) were used to construct the de novo‐based repeat library. RepeatMasker (v4.1.1) (Tarailo‐Graovac and Chen, 2009) was used to mask the genome and annotate the TE elements using the library combined by RepeatModeler, LTR_retriever, and Repbase (v15.02). TEsorter (Zhang et al., 2022) was used to perform superfamily‐level classification of intact LTR retrotransposons. Tandem repeats were identified using Tandem Repeats Finder (v4.07b) (Benson, 1999).

The protein‐coding genes were predicted using a combination of three methods: *de novo* gene prediction, homology‐based gene prediction, and transcriptome‐based gene prediction as described previously (He et al., 2023). For *de novo* prediction, Augustus (v3.2.3) (Stanke and Waack, 2003) was employed, while Genomethreader (v1.7.3) (Gremme et al., 2005) was used for homology‐based gene predictions. For transcriptome‐based predictions, Trinity (v2.12) (Haas et al., 2013) was used to assemble all the RNA‐seq data, followed by further alignment to the genome with PASA (v2.3) (Haas et al., 2003) software. In addition, the RNA‐seq reads were mapped to the genome using HISAT2 (v2.2.1) (Kim et al., 2019), and the resulting reads were assembled into transcripts using StringTie (v2.1.6) (Pertea et al., 2015). Subsequently, the assembled transcripts were used to predict the longest ORFs using TransDecoder (v5.1.0) (https://github.com/TransDecoder/). All evidence of gene models was merged into a non‐redundant set using EvidenceModeler (Haas et al., 2008). Based on the same prediction pipeline, we re‐annotated the previously published genome of the hexaploid cultivated oats, Sanfensan. For functional annotation, the protein sequences were searched against various databases using BLASTP with an e‐value threshold of 1e‐5. This search included InterProScan (v5.48), the Swiss‐Prot databases, and the NR database. Additionally, GO annotations and Pfam domain annotations were performed using InterProScan, while KEGG orthology (KO) terms were assigned through homology searches with KofamScan (Aramaki et al., 2020).

### Subgenome partitioning

Three distinct approaches were utilized to partition the *A. barbata* genome into subgenomes. First, the genomic sequence of *A. strigosa* (Li et al., 2021), characterized by the AA genome, was aligned to the *A. barbata* genome using MUMmer (v4.0.0) (Marcais et al., 2018) and two subgenomes were identified based on sequence similarity. Second, protein‐coding genes from the two *A. barbata* subgenomes, in conjunction with those from *A. strigosa* and Sanfensan‐D, were analyzed to define orthogroups utilizing OrthoFinder (v2.3.14) (Emms and Kelly, 2019). The single‐copy gene clusters shared by them were extracted and aligned for each group of homologous chromosomes using MUSCLE (v3.8.1551) (Edgar, 2004), and then selected to construct phylogenetic trees. Third, subgenome‐specific *k*‐mers were identified using SubPhaser (Jia et al., 2022) to allocate chromosomes to the two subgenomes.

To verify whether *A. barbata* is an allopolyploid or an autopolyploid species, we collected genome and annotation information for allopolyploid species (cotton, wheat, peanut) and autopolyploid species (sugarcane, potato, alfalfa). We used MUMmer to perform alignments between subgenomes of different species and calculated the subgenome similarity for both allopolyploids and autopolyploids. Additionally, we used MCScanX with default parameters to identify collinear homologous regions with paralogous gene pairs between subgenomes. Multiple protein‐coding DNA alignments were constructed using ParaAT (v2.0) (Zhang et al., 2012) with the parameters (‐m clustalw2 ‐f axt), and the synonymous substitution rate (*K*_*s*_) values for each homologous gene pair were calculated using *KaKs*_Calculator (v2.0) (Wang et al., 2010) with default parameters.

### Gene families and phylogenetic analysis

Comparative genomic and phylogenetic analyses were conducted on the genomes of six *Avena* species (*Avena strigosa*, *Avena atlantica*, *Avena longiglumis*, *Avena sativa* ssp. *nuda* cv. Sanfensan, *Avena insularis*, *Avena eriantha*), with the genomes of *Triticum aestivum* and *Secale cereale* as outgroups. Across the 15 subgenomes of the nine species, single‐copy genes were identified using the OrthoFinder (v2.3.14) pipeline with default parameters. These genes were aligned into a concatenated amino acid sequence using MUSCLE (v3.8.1551). The best‐fit model was predicted using ProtTest3 (Darriba et al., 2011), and a maximum likelihood tree was constructed using RAxML (v8.0.0) (Stamatakis, 2014) with the PROTGAMMAIJTTF model and 100 bootstrap replicates. Divergence times were estimated with MCMCTree program from the PAML (v4.7) (Yang, 2007) package and corrected based on the timeline in the TIMETREE database (http://www.timetree.org/). To further verify the time of polyploidization, the divergence time was estimated using the distribution of *Ks* of the homologous genes with the formula *T* = *K*_*s*_/2 *μ*, in which *T* is the divergence time, *K*_*s*_ is the peak value of the synonymous substitution rate and *μ* is the synonymous mutation rate for *Avena* species (6.5 × 10^−9^) as described previously. The *Ks* was calculated by *K*_*a*_ *K*_*s*_‐Calculator (v2.0). Gene family contractions and expansions in each (sub)genome were identified using CAFÉ (v4.2.1) (Han et al., 2013) with default parameters. Collinear blocks were obtained using JCVI (v1.1.19, MCscan for Python) (Wang et al., 2012) with the default setting. Functional enrichment analysis for GO and KEGG was conducted using the R package *clusterProfiler*.

### Identification of disease‐resistance genes

The putative resistance genes (R‐genes) were identified using the RGAugury pipeline (Li et al., 2016), employing default settings. Concurrently, genes conferring resistance to powdery mildew, rust, and smut diseases, which had been cloned from Poaceae species, were obtained from NCBI. The BLASTP algorithm was then applied to scan both the *A. barbata* and Sanfensan genomes to detect orthologous genes corresponding to these disease‐resistance genes, with a stringent threshold of identity ≥ 50% and coverage ≥ 50%.

### The evolution and polyploidization history of *A. barbata*

To infer the ancestral donors of the A and B subgenomes in *A. barbata*, we downloaded whole‐genome sequencing data for 13 *Avena* species (Table S6), representing the genome types found among extant *Avena* diploid, tetraploid, and hexaploid species. The raw sequence reads from polyploids were aligned to the *A. barbata* and Sanfensan genomes using BWA with default settings. Reads that mapped uniquely were isolated using SAMtools and allocated to the A, B, C, and D subgenomes. Following this, the reads derived from both diploid species and separated polyploid were mapped to the A and B subgenomes of *A. barbata* using BWA. Then, the identity and coverage were calculated using a 5 Mb window.

Additionally, these reads were mapped to the A subgenome of Sanfensan. Genomic variants were detected using the HaplotypeCaller module in GATK (v4.1.9.0) (McKenna et al., 2010), and the filtering criteria for SNP are as follows: “DP ≥ 40 & & MQ ≥ 30 & & %QUAL > 40.” The SNPs were employed to construct a maximum likelihood (ML) tree using the RAxML software and performing 100 bootstrap replicates.

In parallel, we downloaded the chloroplast genomes of 15 *Avena* species, *Lolium perenne*, and *Triticum aestivum*. Multiple sequence alignment was carried out using MAFFT, and the resulting alignment was fed into IQ‐TREE (v.2.0.6) (Minh et al., 2020) with the parameters “‐st DNA ‐bb 1000.”

### Identification of structural variation

Genome alignment was performed using the MUMmer program with parameters “–mum –mincluster 1000 –minmatch 100.” Single nucleotide polymorphisms and indels were identified by running show‐SNPs. Then, the SyRI pipeline (Goel et al., 2019; Qin et al., 2021) was used to identify SVs (insertions, deletions, translocations, and inversions) among three genomes. We then retained SVs with variant sizes of over 50 bp.

### Asymmetric evolution of *A. barbata* subgenomes

The raw reads from various tissue RNA samples were filtered using Fastp (v0.20.1) (Chen et al., 2018). Subsequently, clean reads were mapped to the reference genome of *A. barbata* using HISAT2 with default settings. The expression levels of individual genes were assessed using StringTie, which provided estimates in transcripts per million (TPM) values. Collinear gene pairs within the A and B subgenomes of *A. barbata* were identified by MCScanX, and only those gene pairs maintaining a 1:1 collinear relationship between the two subgenomes were retained. An analysis of the HEB was carried out on syntenic gene pairs across the subgenomes. DESeq2 was used for differential expression analysis. Gene pairs showing more than a two‐fold difference in expression across all tissues were classified as dominant gene pairs.

Genes that were unpaired within the syntenic blocks between subgenomes potentially signified the deletion of their homoeologs in the complementary subgenome. Then, the unpaired genes were used as queries to search for their homoeologs within the syntenic block of the opposite subgenome, aiming to identify gene deletion. Blastp software was used with thresholds of e‐value < 10e−5, similarity ≥ 50% and coverage > 50%.

### Genotyping by sequencing (GBS)

GBS data from 211 *A. barbata* samples, each derived from distinct ecological niches, were downloaded from the NCBI Short Read Archive under the project number PRJNA781854 (Table S13). After trimming and quality filtering, the reads were mapped to the *A. barbata* reference genome using BWA. Reads with low mapping quality (MQ < 30) were filtered using SAMtools. Genetic variants were then detected using the Genome Analysis Toolkit (GATK) 4.0 with default parameters. Population structure was inferred using Admixture (v1.3.0) (Alexander and Lange, 2011) with cluster numbers (K) ranging from 1 to 5. A phylogenetic tree was constructed using FASTME 2.0 (www.atgc-montpellier.fr/fastme/) based on a P‐distance matrix calculated by VCF2Dis (v1.46; https://github.com/BGI-shenzhen/VCF2Dis). To explore whether one population was admixed by the other two, we performed the *F3* test using “qp3Pop” from ADMIXTOOLS (v5.1).

### Transcriptome and qRT‐PCR

Primers for qRT‐PCR (Table S15) were designed with Primer Premier 5.0. Total RNA was extracted from different tissues of the collected samples using the RNeasy Plant Mini Kit (Cat. No. 74903, Qiagen, Hilden, Germany) and reverse transcribed into first‐strand cDNA using the PrimeScript^TM^ RT reagent kit with gDNA Eraser (Cat. No. RR047A, TaKaRa, Shiga, Japan) according to the manufacturer's instructions. Quantitative RT‐PCR assays were conducted with TB Green® Premix Ex Taq™ (Tli RNaseH Plus) (Cat. No. RR420A; TaKaRa, Shiga, Japan) on the Bio‐Rad Real‐time PCR System (Bio‐Rad, Hercules, CA, USA). The PCR program was 95°C for 3 min, and then 40 cycles of 95°C for 10 s, annealing temperature (Tm) for 10 s, and 72°C for 20 s. Expression levels were calculated using the 2^−ΔΔCT^ method.

## CONFLICTS OF INTEREST

The authors declare no conflict of interest.

## AUTHOR CONTRIBUTIONS

H.D. conceived and supervised the project; Q.H. and H.D. wrote the paper; Y.X. sequenced and processed the raw data; Q.H. and Y.X. assembled and annotated the genome; Q.H., Y.X., and Ya.W. performed the phylogenetic and genome evolution analysis; Q.H., Ya.W., and Yi.W. conducted the transcriptome analysis; Q.H., W.L., T.L., and Ya.W. performed population analyses. T.L., N.L., and Q.H. conducted qRT‐PCR. Z.G. offered invaluable guidance. All authors read and approved this manuscript.

## Supporting information

Additional Supporting Information may be found online in the supporting information tab for this article: http://onlinelibrary.wiley.com/doi/10.1111/jipb.13902/suppinfo

**Figure S1.** The genome size estimation of *Avena barbata*
**Figure S2.** Genome assembly and quality assessment
**Figure S3.** Collinear gene pairs between subgenome A and B
**Figure S4.** Identification of *Avena barbata* centromere based on Chip‐seq peak, gene density, long terminal repeat (LTR) density, and *k*‐mer frequency with 10 Mb window
**Figure S5.** The comparative analysis of centromere region between two subgenomes
**Figure S6.** Disease‐resistance genes identified in different subgenomes
**Figure S7.** Identification and distribution of structural variation
**Figure S8.** Size distribution of deletion, insertion, inversion and translocation
**Figure S9.** Verification of translocation using Hi‐C heatmaps and the distribution of HiFi reads
**Figure S10.** Verification of deletion, insertion, and inversion
**Figure S11.** Expression and functional enrichment analysis of genes affected by PAV
**Figure S12.** Subgenome dominance analysis
**Figure S13.** Plot of gene retention rates within synteny blocks across two subgenomes
**Figure S14.** Gene Ontology (GO) enrichment analysis for lost genes in A subgenome and B subgenome
**Figure S15.** RNA‐seq analysis of the plant aerial portion at 0, 12, and 24 h post‐PEG6000 treatment
**Figure S16.** Enrichment analysis of genes with persistent differential expression following drought stress
**Figure S17.** Confirmation of the expression patterns between quantitative real‐time polymerase chain reaction (qRT‐PCR) and transcriptome at 0, 12, and 24 h post‐PEG6000 treatment

**Table S1.** Summary of the sequencing data for *Avena barbata*
**Table S2.** Telomere sequences of *A. barbata* genome
**Table S3.** Statistics for transposable elements in the *A. barbata* genome
**Table S4.** Gene structure annotation
**Table S5.** Gene functions annotation
**Table S6.** Locations and length of centromeres in *A. barbata* genome
**Table S7.** The genomes resequencing data of different *Avena* species
**Table S8.** The chloroplast genome information used in this study
**Table S9.** Number of intact long terminal repeat (LTR) in five subgenome
**Table S10.** The number of structural variations among different subgenomes
**Table S11.** Length of structural variations among different subgenomes
**Table S12.** The proportion of different types of long terminal repeat (LTR) related to structural variation (SV)
**Table S13.** The agronomically important genes associated with B subgenome‐specific structural variation (SV)
**Table S14.** GBS data information of 211 *A. barbata* accessions
**Table S15.** F3‐Statistics result
**Table S16.** Functional annotation of genes containing single nucleotide polymorphisms (SNPs) that distinguish between two ecotypes

## Data Availability

For convenient and effective use of this genome resource, the raw sequencing data and genome assembly were deposited in the Chinese National Genomics Data Center database (https://bigd.big.ac.cn/) under the BioProject accession numbers PRJCA027164. Source data are provided in this article and the associated supplementary materials.
