# Supplementary material for: High‐quality genome of allotetraploid Avena barbata provides insights into the origin and evolution of B subgenome in Avena
Source: J Integr Plant Biol. 2025 Apr 14;67(6):1515–32. doi: 10.1111/jipb.13902 (PMC12131679; doi:10.1111/jipb.13902)
Supplement: Supplementary file 1 — Figure S1. The genome size estimation of Avena barbata Figure S2. Genome assembly and quality assessment Figure S3. Collinear gene pairs between subgenome A and B Figure S4. Identification of Avena barbata centromere based on Chip‐seq peak, gene density, long terminal repeat (LTR) density, and k‐mer frequency with 10 Mb window Figure S5. The comparative analysis of centromere region between two subgenomes Figure S6. Disease‐resistance genes identified in different subgenomes Figure S7. Identification and distribution of structural variation Figure S8. Size distribution of deletion, insertion, inversion and translocation Figure S9. Verification of translocation using Hi‐C heatmaps and the distribution of HiFi reads Figure S10. Verification of deletion, insertion, and inversion Figure S11. Expression and functional enrichment analysis of genes affected by PAV Figure S12. Subgenome dominance analysis Figure S13. Plot of gene retention rates within synteny blocks across two subgenomes Figure S14. Gene Ontology (GO) enrichment analysis for lost genes in A subgenome and B subgenome Figure S15. RNA‐seq analysis of the plant aerial portion at 0, 12, and 24 h post‐PEG6000 treatment Figure S16. Enrichment analysis of genes with persistent differential expression following drought stress Figure S17. Confirmation of the expression patterns between quantitative real‐time polymerase chain reaction (qRT‐PCR) and transcriptome at 0, 12, and 24 h post‐PEG6000 treatment [file JIPB-67-1515-s001.docx]

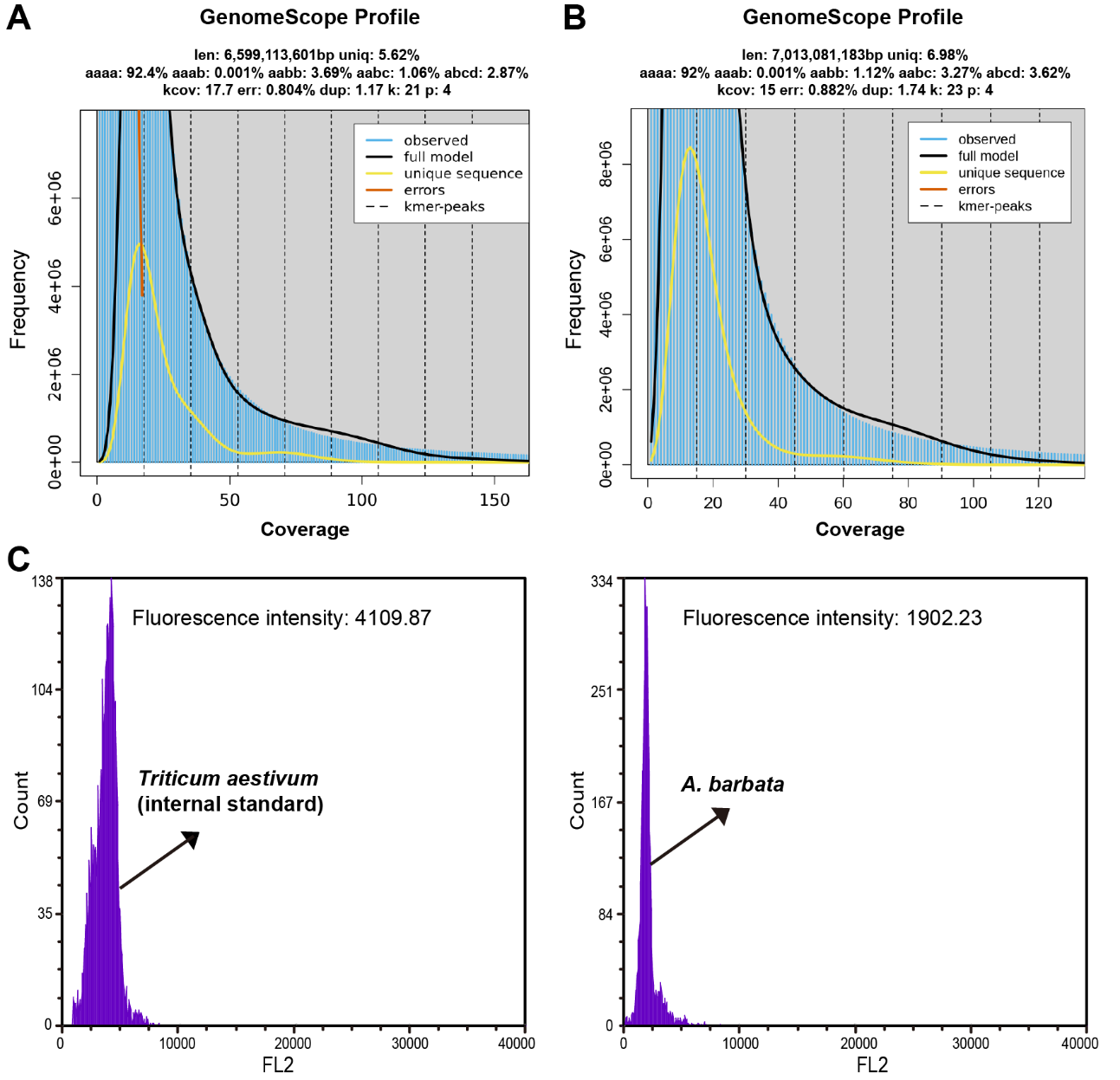


**Figure S1.** The genome size estimation of *Avena barbata*. **(A)** Estimation of genome size using GenomeScope with Illumina sequencing data. **(B)** Estimation of genome size using GenomeScope with HiFi reads. **(C)** Estimation of genome size by flow cytometry with *Triticum aestivum* as the internal standard.


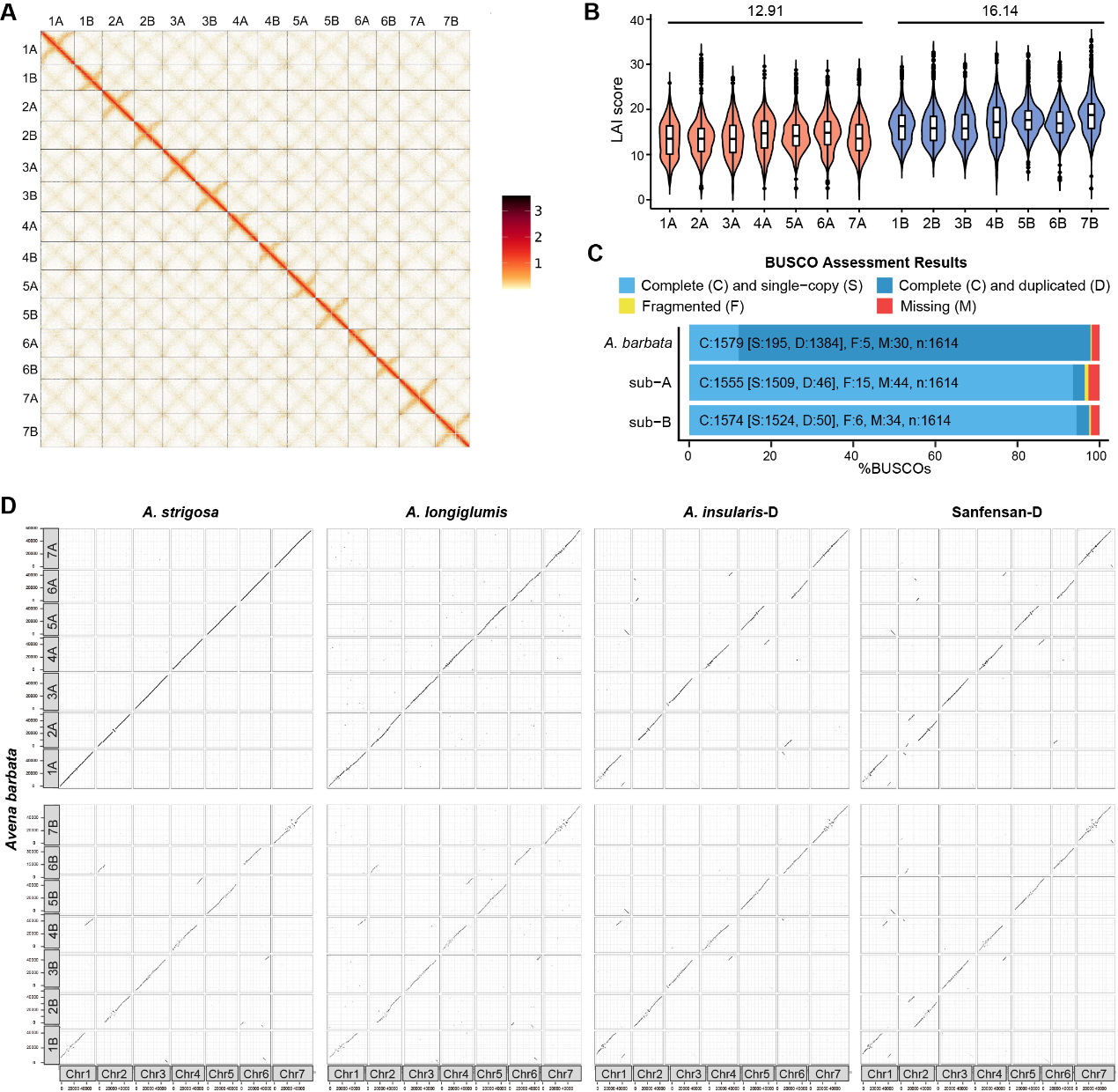


**Figure S2. Genome assembly and quality assessment.**

**(A**) Hi–C interactions between the eight chromosomes of subgenome A (A1–A7) and eight chromosomes of subgenome B (B1–B7). **(B)** LTR Assembly Index (LAI) score for each chromosome of *A. barbata*. **(C)** BUSCO completeness assessment for genomics data of *A. barbata*. **(D)** Genome-wide synteny analysis among *A. barbata*-A, *A. barbata*-B, *A. strigosa*, *A. longiglumis*, *A. insularis*-D, and Sanfensan-D.


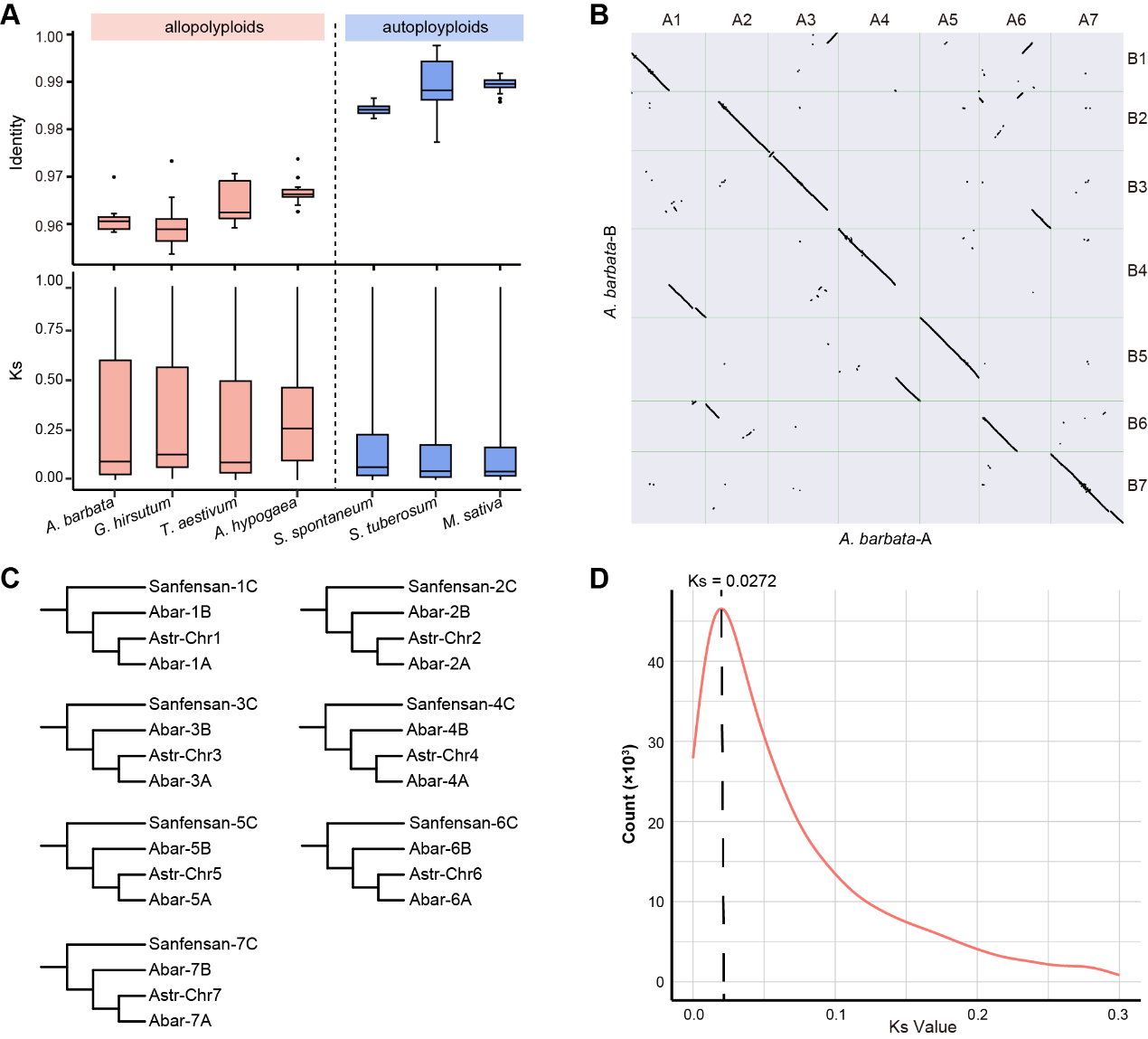


**Figure S3. Collinear gene pairs between subgenome A and B.**

**(A)** Top is Sequence identity distribution of homologous chromosome blocks and Ks distributions of gene pairs among different monoploid genomes for *A. barbata*, *Gossypium hirsutum*, *Triticum aestivum*, *Arachis hypogaea*, *Saccharum spontaneum*, *Solanum tuberosum*, and *Medicago sativa*. **(B)** The visualization of pairwise synteny between *A. barbata* subgenomes A and B. **(C)** The partition and phasing of *A. barbata* subgenomes using a phylogenetic tree constructed from orthologous genes. **(D)** Distribution of synonymous substitution rates (*Ks*) between subgenomes A and B indicates polyploidization time.


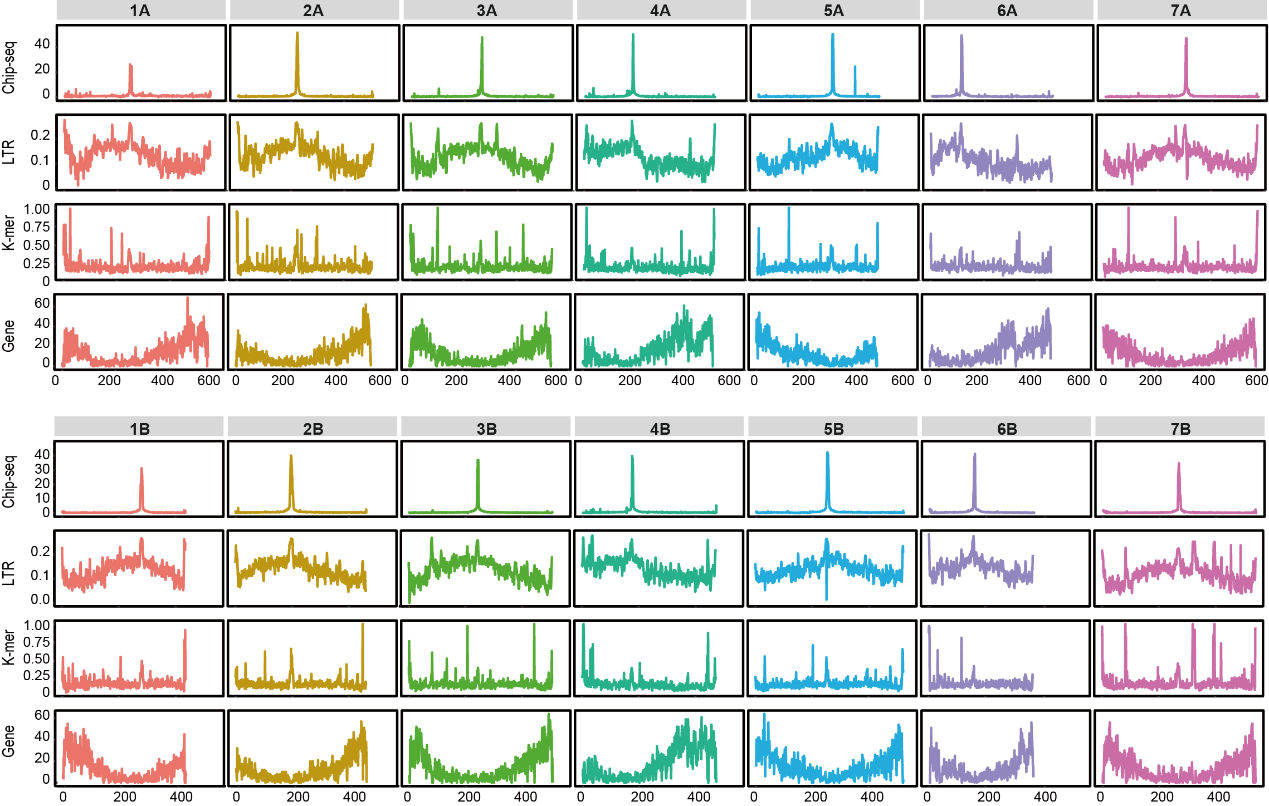


**Figure S4. Identification of *A. barbata* centromere based on Chip-seq peak, gene density, LTR density, and *k*-mer frequency with 10 Mb window.**


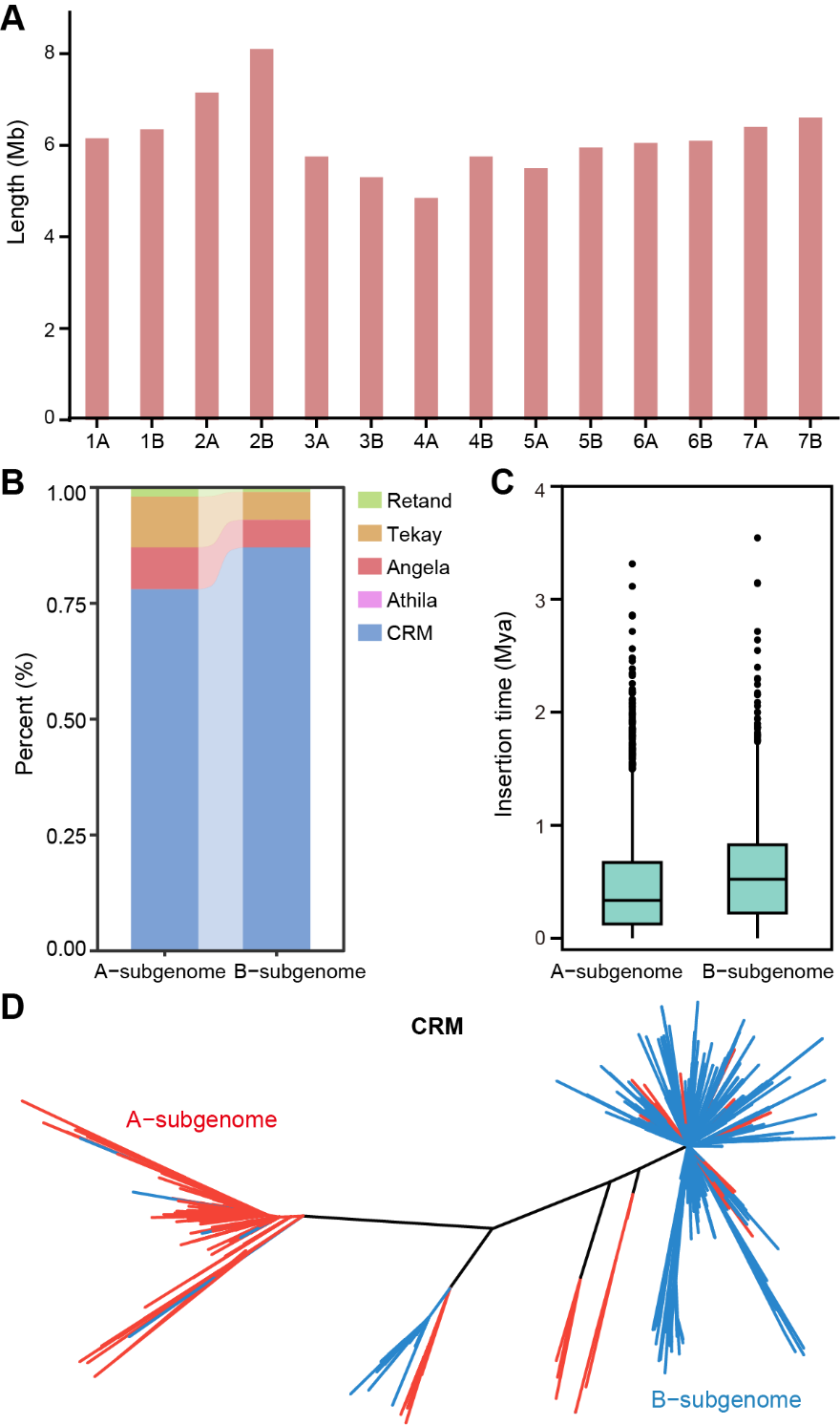


**Figure S5.** **The comparative analysis of centromere region between two subgenomes. (A)** The centromere lengths on the 14 chromosomes of *A. barbata*. **(B)** Proportion of LTR-RT family in centromere regions of different subgenome in *A. barbata* genome. **(C)** The insertion time of LTR‐RT family for the centromeres region in *A. barbata* genome. **(D)** A phylogenetic tree constructed using the RT domain sequences of CRM retrotransposons within the centromeric region. The branches are colored red for the A subgenome and blue for the B subgenome.


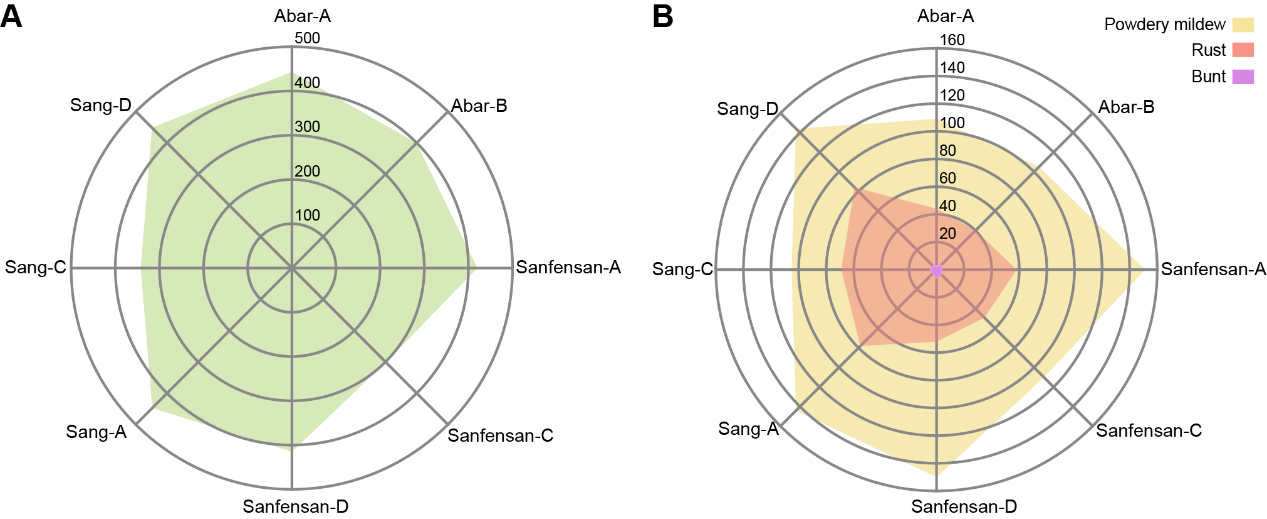


**Figure S6. Disease resistance genes identified in different subgenome.**

**(A)** Comparison of the number of predicted R-genes identified in the subgenomes of *A. barbata* and two hexaploid cultivated oat. **(B)** Predicted number of cloned resistance genes against powdery mildew, rust, and black smut in the subgenomes of *A. barbata* and two hexaploid cultivated oat.


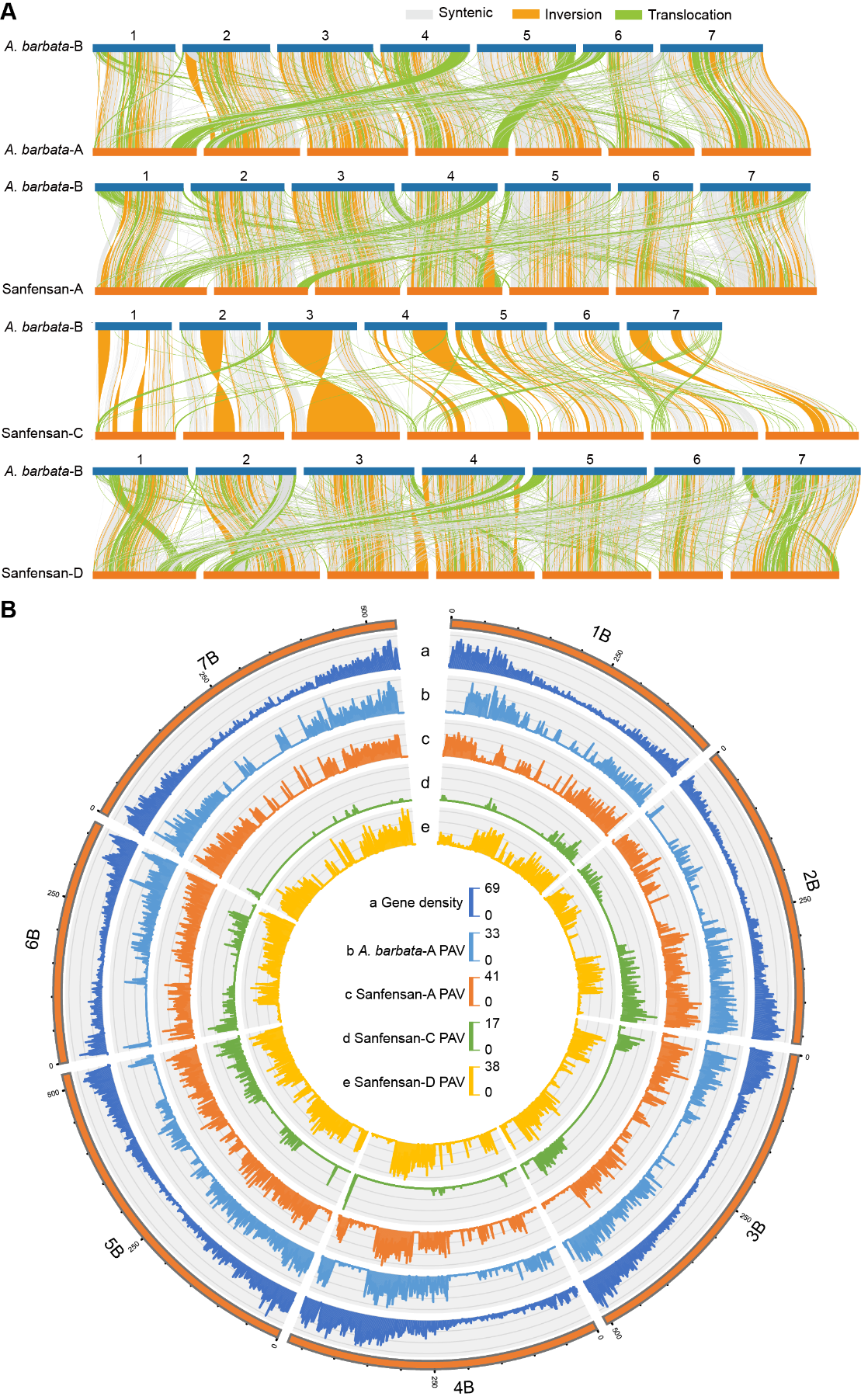


**Figure S7. Identification and distribution of structural variation.**

**(A)** Structural variations for five subgenomes using *A. barbata* subgenome B as reference with Syri software. **(B)** Distribution of SVs of four subgenomes across the 7 chromosomes of *A. barbata* subgenome B.


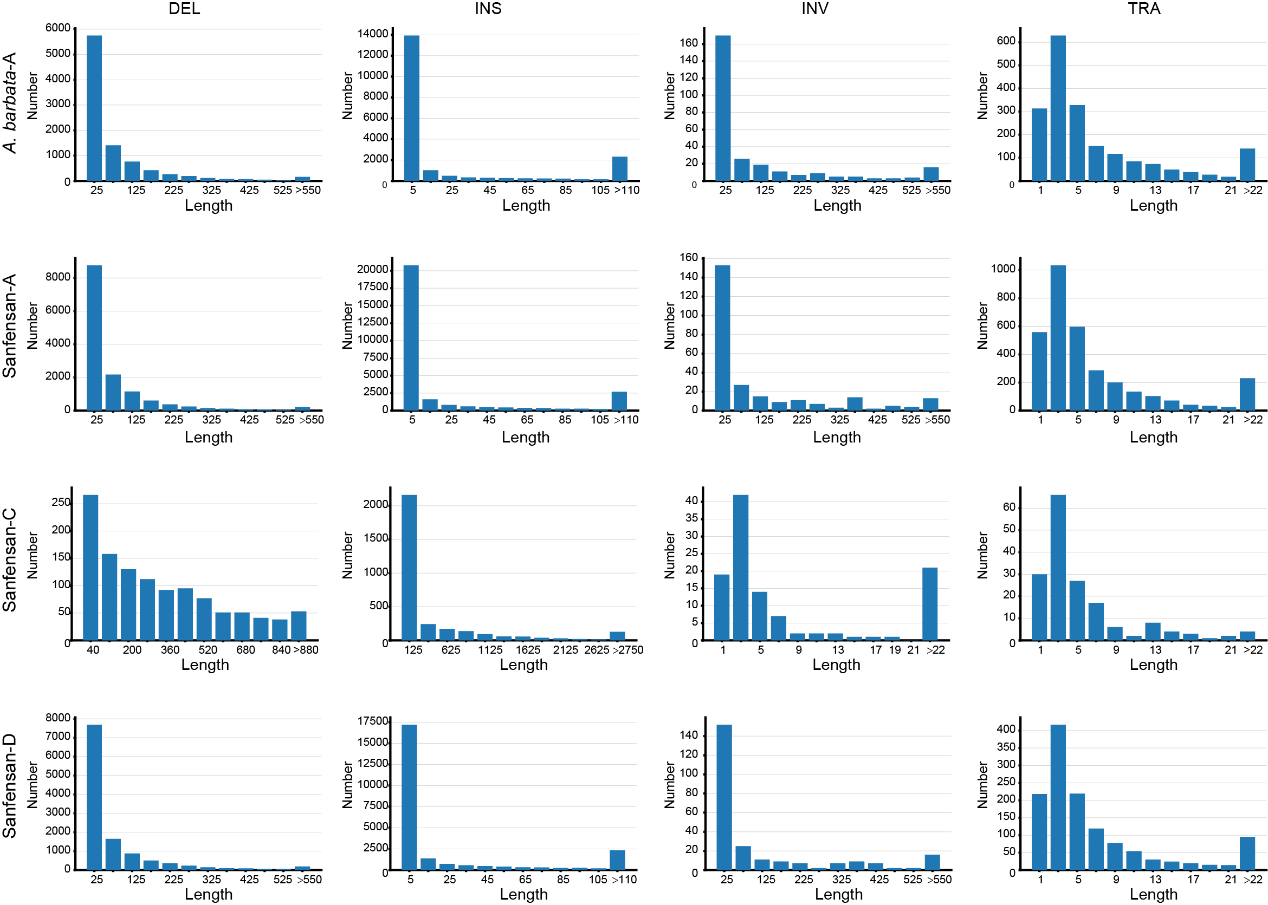


**Figure S8. Size distribution of deletion (DEL), insertion (INS), inversion (INV) and translocation (TRA) in *A. barbata*-A, Sanfensan-A, Sanfensan-C, and Sanfensan-D subgenome related to *A. barbata*-B subgenome.**


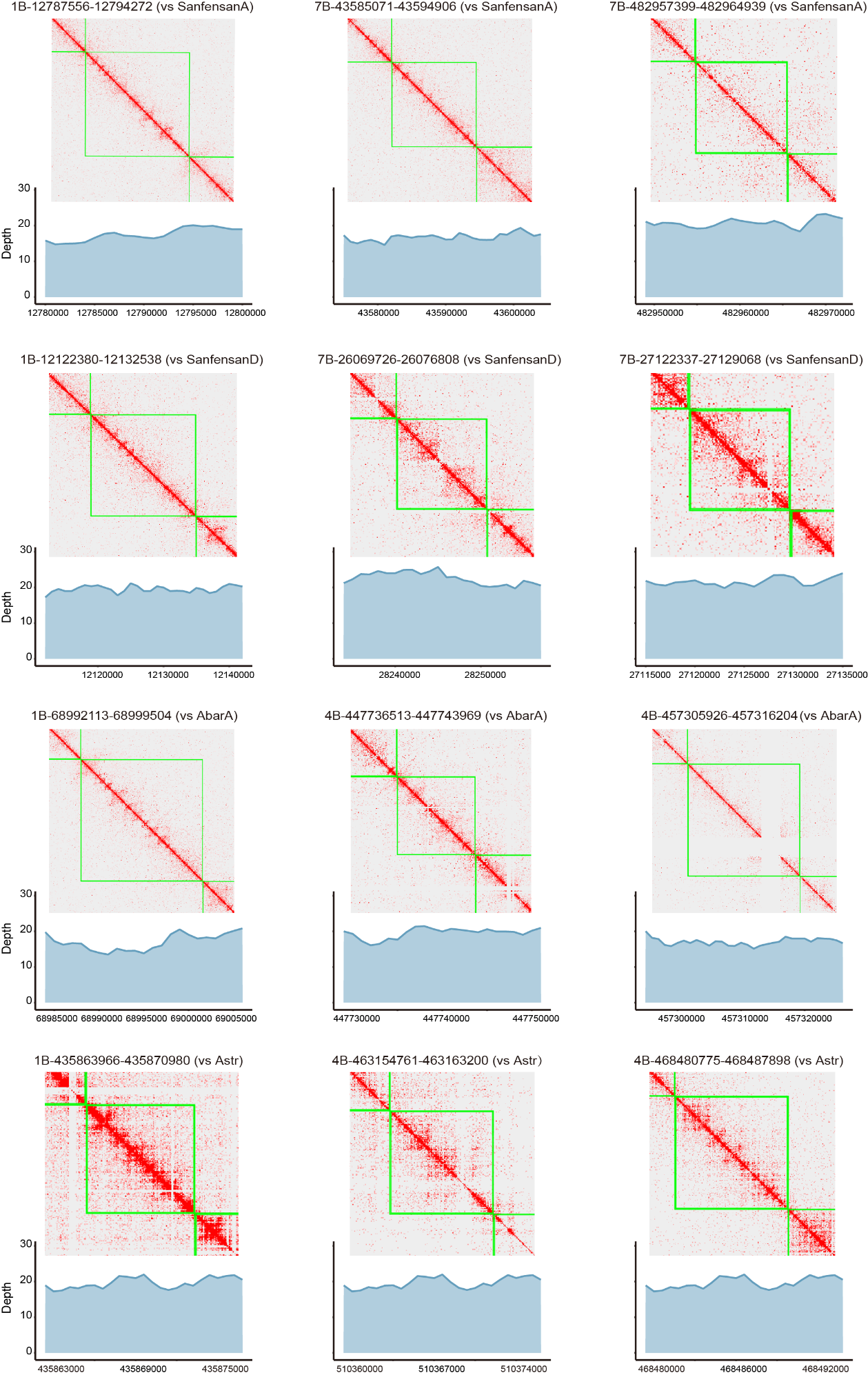


**Figure S9. Verification of translocation using Hi-C heatmaps and the distribution of HiFi reads.**


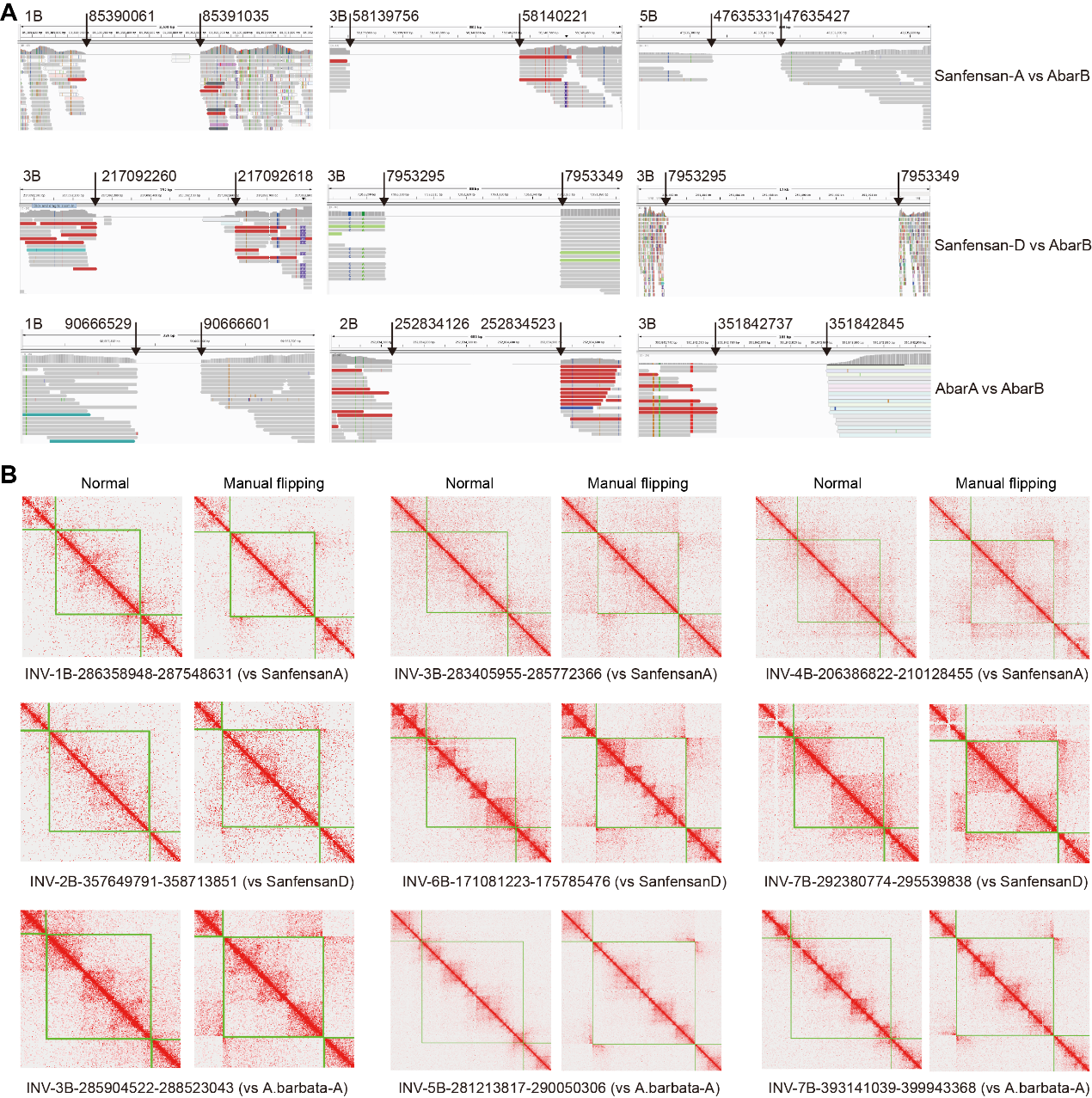


**Figure S10. Verification of structural variation.** **(A)** The short-read data were used to validate the borders of deletions randomly selected between B subgenome and other subgenome. **(B)** Illustration of inversion identified between B subgenome and other subgenome by Hi-C contact map. Chromatin interaction heatmap revealed inversion signals appearing after manual flipping


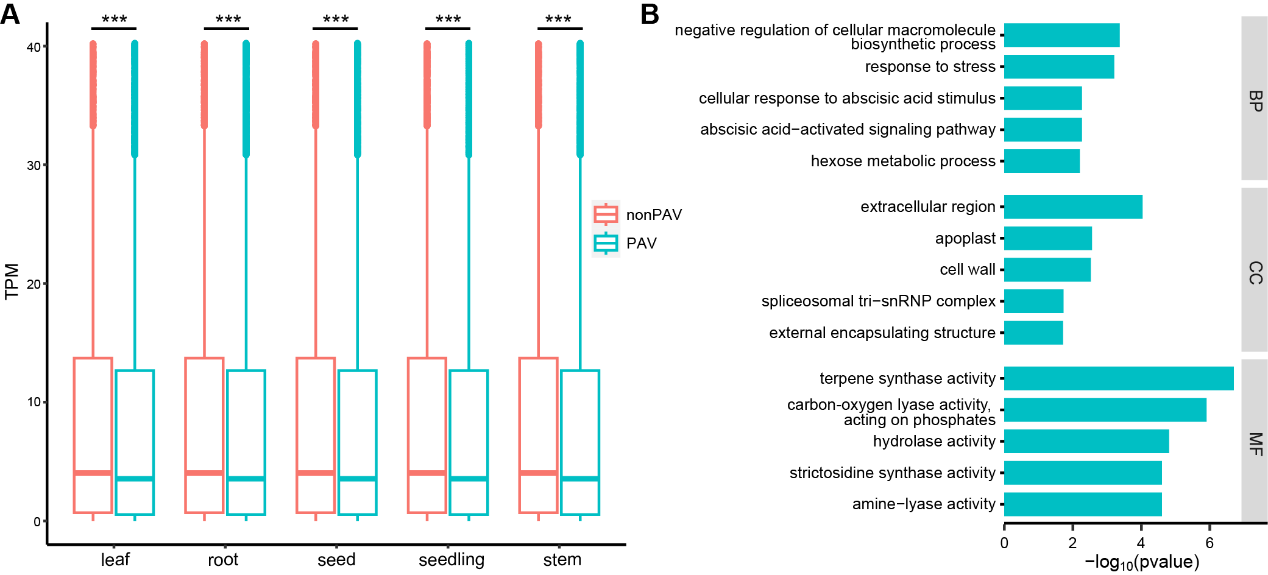


**Figure S11. Expression and functional enrichment analysis of genes affected by PAV.**

**(A)** Expression level of PAV associated and non-PAV gene in different tissues (Two-tailed Student’s t test, ****P* < 0.001)**. (B)** GO enrichment analysis of genes influenced by PAV in *A. barbata* subgenome B.


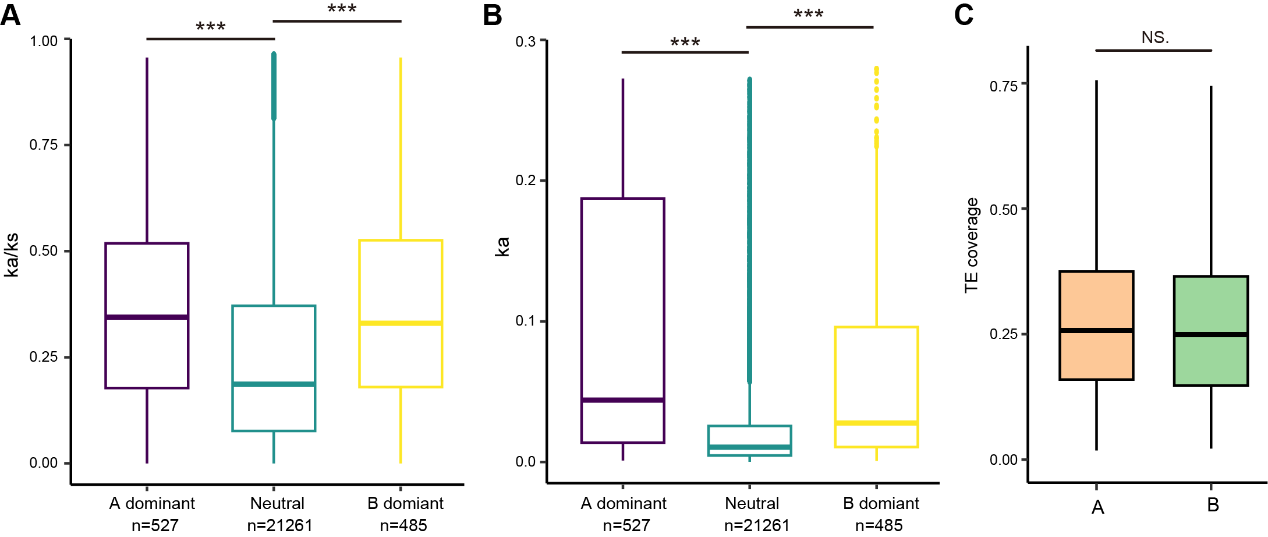


**Figure S12. Subgenome dominance analysis.**

The *Ka/Ks* **(A)** and *Ka* **(B)** of the subgenome A/B dominant genes and neutrally expressed genes. **(C)** Repetitive element coverage of the dominant genes region in subgenome A/B.


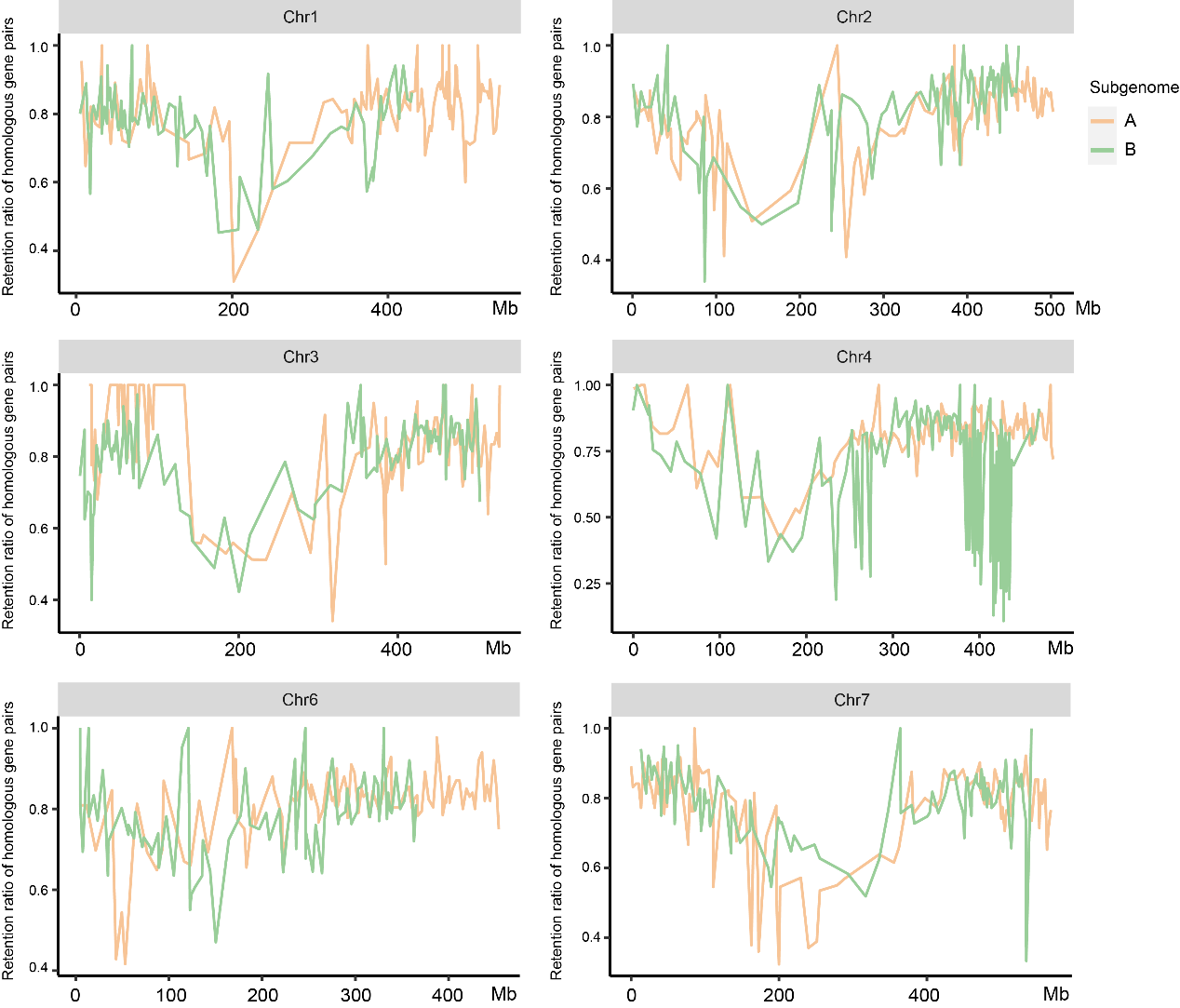


**Figure S13. Plot of gene retention rates within synteny blocks across two subgenomes.**


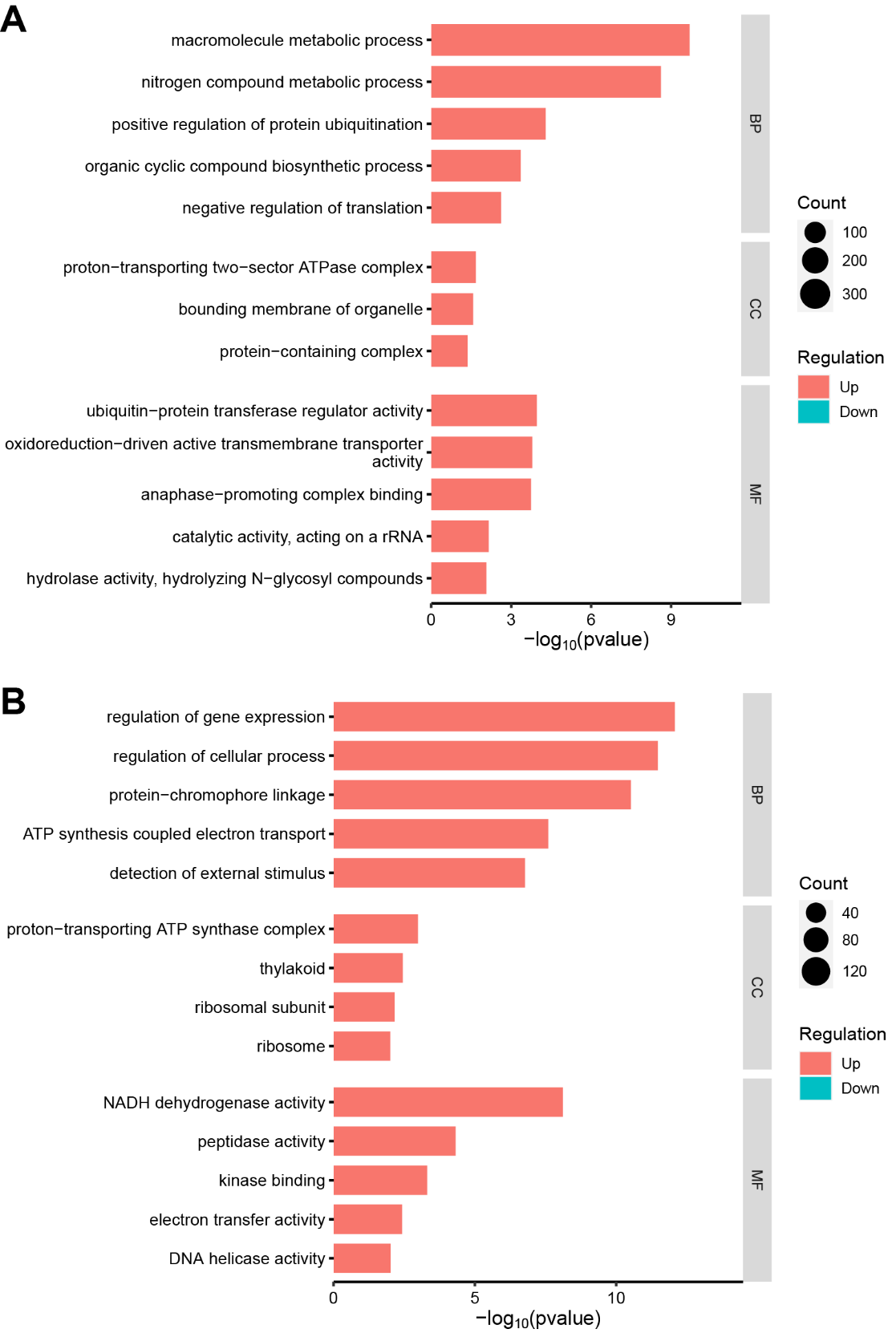


**Figure S14. Gene Ontology (GO) enrichment analysis for lost genes in A subgenome (A) and B subgemome (B).**


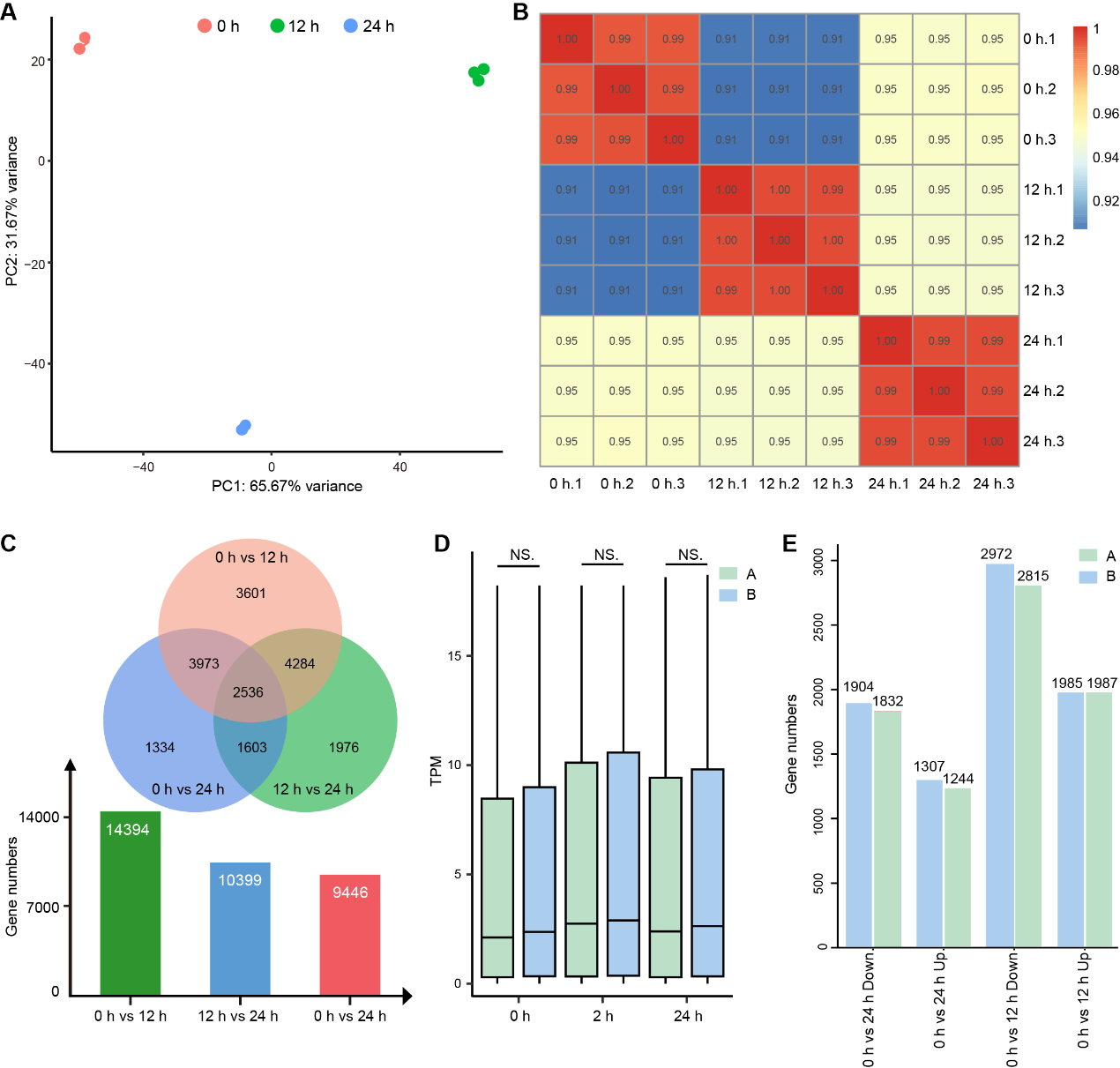


**Figure S15. RNA-seq analysis of the plant aerial portion at 0 h, 12 h, and 24 h post-PEG6000 treatment.**

**(A)** The PCA map of RNA-seq samples. (**B)** Correlation matrix of RNA-seq samples. **(C)** The number of differentially expressed genes under different treatments time. **(D)** Boxplot showing expression levels of all the expressed genes (FPKM ≥ 0.1) across different treatments time in the A and B subgenomes of *A. barbata*. NS: no significant difference. **(E)** The numbers of up-regulated and down-regulated expression gene under different treatments time in the A and B subgenomes of *A. barbata*.


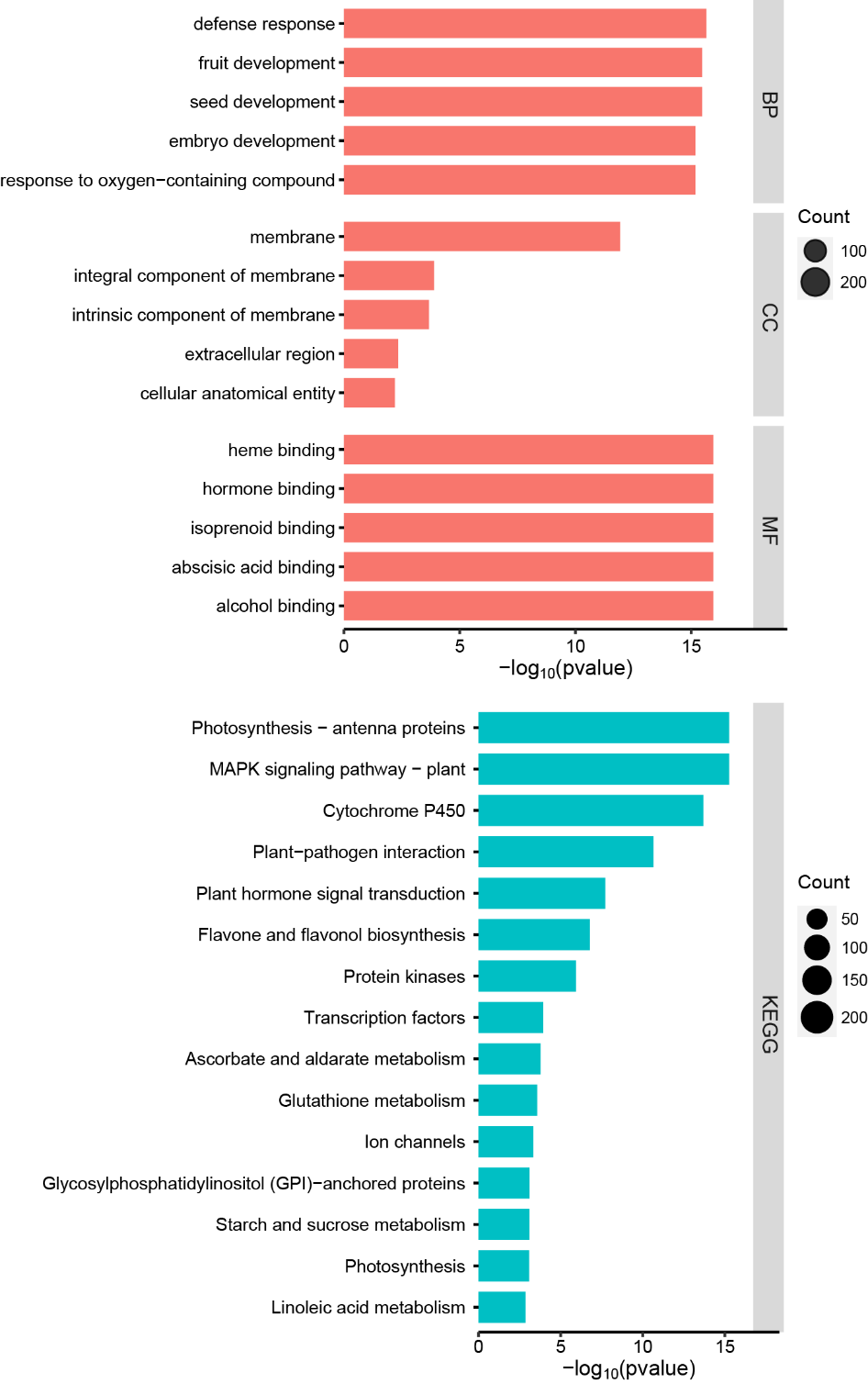


**Figure S16. Enrichment analysis of KEGG (Kyoto Encyclopedia of Genes and Genomes) and Gene Ontology (GO) terms for genes with persistent differential expression following drought stress.**


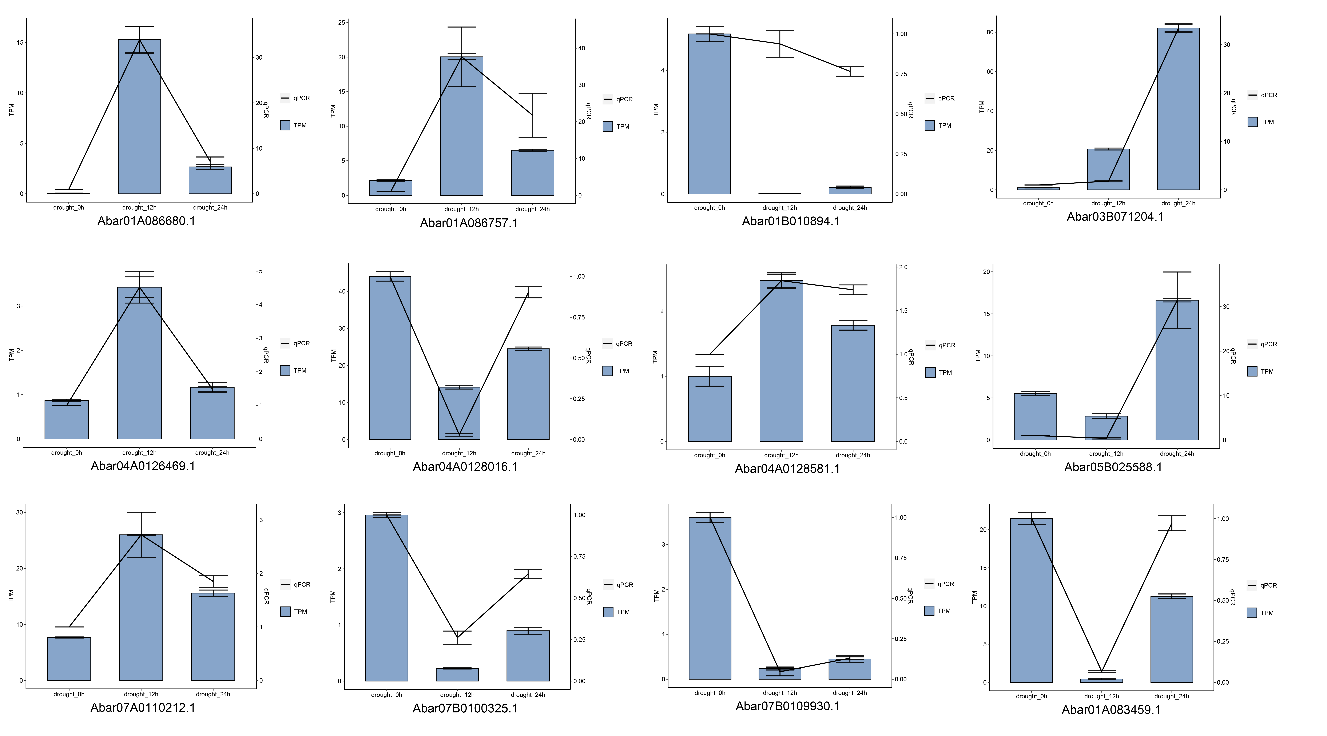


**Figure S17.** **Confirmation of the expression patterns between qRT-PCR and transcriptome at 0 h, 12 h, and 24 h post-PEG6000 treatment.**
