# Supplementary material for: High‐quality genome of allotetraploid Avena barbata provides insights into the origin and evolution of B subgenome in Avena
Source: J Integr Plant Biol. 2025 Apr 14;67(6):1515–32. doi: 10.1111/jipb.13902 (PMC12131679; doi:10.1111/jipb.13902)
Supplement: Supplementary file 2 — Table S1. Summary of the sequencing data for Avena barbata Table S2. Telomere sequences of A. barbata genome Table S3. Statistics for transposable elements in the A. barbata genome Table S4. Gene structure annotation Table S5. Gene functions annotation Table S6. Locations and length of centromeres in A. barbata genome Table S7. The genomes resequencing data of different Avena species Table S8. The chloroplast genome information used in this study Table S9. Number of intact long terminal repeat (LTR) in five subgenome Table S10. The number of structural variations among different subgenomes Table S11. Length of structural variations among different subgenomes Table S12. The proportion of different types of long terminal repeat (LTR) related to structural variation (SV) Table S13. The agronomically important genes associated with B subgenome‐specific structural variation (SV) Table S14. GBS data information of 211 A. barbata accessions Table S15. F3‐Statistics result Table S16. Functional annotation of genes containing single nucleotide polymorphisms (SNPs) that distinguish between two ecotypes [file JIPB-67-1515-s002.pdf]

Supplementary Table S1. Summary of the sequencing data for *A. barbata*.

Sample	Sequence Type	Raw data (Gb)	Length (bp)	Tissue	Alignment Rate
Avena barbata	**HiFi**	134.66	N50:19642	Leaf	99.92%
	**Hi-C**	310.28	2×150	Leaf	99.71%
	**Illumina paired-end reads**	120.94	2×150	Leaf	99.89%
	**RNA-seq**	13.95	2×150	Root_1	80.21%
	**RNA-seq**	12.94	2×150	Root_2	80.34%
	**RNA-seq**	14.08	2×150	Root_3	80.09%
	**RNA-seq**	14.39	2×150	Leaf_1	97.56%
	**RNA-seq**	12.08	2×150	Leaf_2	97.55%
	**RNA-seq**	12.10	2×150	Leaf_3	97.44%
	**RNA-seq**	14.03	2×150	stem_1	95.21%
	**RNA-seq**	12.90	2×150	stem_2	94.92%
	**RNA-seq**	13.51	2×150	stem_3	95.24%
	**RNA-seq**	13.47	2×150	seed_1	96.70%
	**RNA-seq**	14.27	2×150	seed_2	96.72%
	**RNA-seq**	12.74	2×150	seed_3	96.66%
	**RNA-seq**	6.86	2×150	seedling1	95.29%
	**RNA-seq**	6.79	2×150	seedling2	94.13%
	**RNA-seq**	6.41	2×150	seedling3	94.11%

Supplementary Table S2. Statistics for telomeres of the *A. barbata* genome.

Chromosomes	Type in Start	Repeat number	Type in End	Repeat number
1A	-	-	TTTAGGG	10949
1B	CCCTAAA	2054	CCCTAAA	39
2A	-	-	-	-
2B	-	-	TTTAGGG	2437
3A	-	-	TTTAGGG	2539
3B	CCCTAAA	694	-	-
4A	CCCTAAA	1953	TTTAGGG	852
4B	CCCTAAA	1671	TTTAGGG	31
5A	-	-	TTTAGGG	4072
5B	CCCTAAA	2197	TTTAGGG	88
6A	CCCTAAA	4137	TTTAGGG	2062
6B	-	-	-	-
7A	-	-	TTTAGGG	5055
7B	CCCTAAA	929	TTTAGGG	1845

Supplementary Table S3. Statistics for transposable elements in the *A. barbata* genome.

	Length （bp）	Percentage of sequence
Total Repeat Fractions	6,045,946,936	87.80%
Class I: Retrotransposon	5,652,461,480	82.08%
LTR Retrotransposon	5,581,982,683	81.06%
Ty1/*Copia*	1,262,826,288	18.34%
Gypsy /DIRS1	2,948,099,651	42.81%
other	1,371,056,744	19.91%
Non-LTR Retrotransposon	263,275,214	3.82%
LINE	70,241,620	1.02%
SINE	237,177	0.00%
Class II: DNA transposon	263,275,214	3.82%
hobo-Activator	2,242,894	0.03%
Tc1-IS630-Pogo	400,859	0.01%
Tourist/Harbinger	16,295,828	0.24%
DNA other	244,228,157	3.55%
unclassified	105,606,271	1.53%

Supplementary Table S4. Statistics of gene structure annotation.

	A. barbata	*A. strigosa*	Sanfensan	Sang
Gene Number	93821	39885	131864	152335
Mean CDS length (bp)	1054	282	984	980
Mean exons per gene	4.0	4.5	3.9	3.9
Mean gene length (bp)	2901	3720	2980	2927
Mean exon length (bp)	264	282	264	384

Supplementary Table S5. Statistics for annotation of gene functions.

	Type	Number
		A. barbata
Annotated	InterPro	63838
	Pfam	56491
	GO	46065
	KEGG	44899
Annotated	\	65910 (70.25%)
Unannotated	\	27911
Total	\	93821

Supplementary Table S6. Locations and length of centromeres in *A. barbata* genome

Chr	Start	End
1A	246750000	252900000
1B	277700000	284050000
2A	218850000	226000000
2B	193650000	201750000
3A	261550000	267300000
3B	240750000	246050000
4A	179150000	184000000
4B	172750000	178500000
5A	276850000	282350000
5B	250650000	256600000
6A	108950000	115000000
6B	155450000	161550000
7A	299350000	305750000
7B	266450000	273050000

Supplementary Table S7. The genomes resequencing data of different *Avena* species.

Species	Genome type	Accession number
A. hirtula	As	SRX6682497
A. wiestii	As	SRX11204926
A. lusitanica	As	SRX6682493
A. strigosa	As	SRX11189678
A. brevis	As	SRX3203310
A. nuda	As	SRX11195168
A. atlantica	As	SRX6682487
A. longiglumis	Al	SRX11248995
A. damascena	Ad	SRX11202819
A. insularis	CD	SRX11322727
A. sativa	ACD	SRX11183453
A. canariensis	ACD	SRX11196842

Supplementary Table S8. The Chloroplast genome information uesd in this study.

Species	Accession	Tpye
T. aestivum	NC_002762.1	Chloroplast
L. multiflorum	NC_019651.1	Chloroplast
A. eriantha	NC_044157.1	Chloroplast
A. clauda	NC_044167.1	Chloroplast
A. canariensis	NC_044161.1	Chloroplast
A. lusitanica	NC_044149.1	Chloroplast
A. damascena	NC_044166.1	Chloroplast
A. longiglumis	NC_044169.1	Chloroplast
A. atlantica	NC_044163.1	Chloroplast
A. nuda	NC_044147.1	Chloroplast
A. strigosa	NC_044171.1	Chloroplast
A. brevis	NC_044172.1	Chloroplast
A. hirtula	NC_050395.1	Chloroplast
A. wiestii	NC_044160.1	Chloroplast
A. insularis	MG674209.1	Chloroplast
A. sativa	NC_027468.1	Chloroplast
A. barbata	NC_044173.1	Chloroplast

Supplementary Table S9. Number of intact LTR in five subgenome.

		A. barbata		**Sanfensan**		
		**A**	**B**	**A**	**C**	**D**
Gypsy	unknown	38106	37744	34802	69254	36837
Copia	unknown	49123	38099	35565	31271	31372
Copia	Angela	16068	13642	14430	12054	12075
Gypsy	Tekay	8250	8987	6692	4742	6870
Gypsy	Retand	7689	6257	5293	7898	6483
Gypsy	CRM	7227	8438	7217	3811	5981
Gypsy	Athila	1366	2183	2042	13275	2670
Copia	Ale	1322	1149	1167	781	1270
Copia	SIRE	1220	918	921	913	1051
Gypsy	Ogre	2337	726	815	61	104
Gypsy	mixture	676	413	198	604	255
Copia	Ikeros	231	355	279	248	356
Copia	TAR	328	265	326	133	303
Gypsy	Reina	253	259	255	248	251
Copia	Bianca	185	201	188	75	165
Gypsy	Galadriel	68	74	79	164	156
Copia	Tork	54	73	115	159	137
Total	-	134763	119733	98660	127055	93645

Supplementary Table S10. The number of structural variations among different subgenomes.

Sample	Structure variations (>=50bp)				
	**Deletion**	**Insertion**	**Inversion**	**Translocation**	**Total**
A. barbata-A vs *A. barbata*-B	9577	7764	358	1969	19668
Sanfensan-A vs *A. barbata*-B	14316	11008	367	3317	29008
Sanfensan-C vs A. barbata-B	1501	1314	157	170	3142
Sanfensan-D vs *A. barbata*-B	12242	10503	346	1301	24392

Sample	Small variations (<50bp)		
	**SNP**	**Indel**	**Total**
A. barbata-A vs *A. barbata*-B	9486654	1158812	10645466
Sanfensan-A vs *A. barbata*-B	12808054	1551748	14359802
Sanfensan-C vs *A. barbata*-B	1350259	233904	1584163
Sanfensan-D vs *A. barbata*-B	14139678	1642063	15781741

Supplementary Table S11. Length of structural variations among different subgenomes.

Sample	Structure variations (>=50bp)				
	Deletions (bp)	Insertions (bp)	Inversions (bp)	Translocations (bp)	Total (bp)
A. barbata-A vs *A. barbata*-B	1,621,751,817	1,552,444,723	557,337,803	19,142,805	3,750,677,148
SanfenSan-A vs *A. barbata*-B	1,782,438,377	1,427,596,282	624,449,758	26,200,556	3,860,684,973
SanfenSan-C vs *A. barbata*-B	1,867,212,465	2,511,051,414	1,026,628,399	931,233	5,405,823,511
SanfenSan-D vs *A. barbata*-B	1,803,140,343	1,575,026,429	606,599,335	11,687,715	3,996,453,822

Supplementary Table S12. The proportion of different types of LTR related to SV

Type	*A. barbata* -A	Sanfensan-A	Sanfensan-D
LTR-*Gypsy*	23.90%	27.33%	30.19%
LTR-*Copia*	12.19%	12.40%	12.76%
LTR-unknown	8.72%	7.59%	7.96%
Unspecified	0.76%	0.80%	0.78%
LINE-L1	1.07%	0.91%	1.09%
DNA-MULE-MuDR	0.12%	0.20%	0.19%
DNA-hAT-Tip100	0.02%	0.02%	0.03%
DNA-CMC-EnSpm	0.73%	0.70%	0.86%
DNA-PIF-Harbinger	0.09%	0.11%	0.16%

Supplementary Table S13. Functional classification of the agronomically important genes associated with B subgenome-specific SV.

Genes name	Matching gene	Matching species	Function
Abar01B013889.1	LOC_Os01g10040	Oryza sativa	Tiller angle
Abar01B013934.1	LOC_Os10g35440	Oryza sativa	Fertility restoration gene
Abar01B013939.1	LOC_Os06g29810	Oryza sativa	Resistance to rice blast
Abar01B015988.1	TraesCS2A02G175700	Triticum aestivum	Grain weight, grain yield per plant
Abar01B017104.1	TraesCS4D02G094400	Triticum aestivum	Drought tolerance
Abar01B017115.1	LOC_Os07g47330	Oryza sativa	Secondary branch number per panicle, grain number, grain length, 1000-grain weight, and the percentage of filled grains
Abar02B029965.1	LOC_Os06g29810	Oryza sativa	Resistance to rice blast
Abar02B031757.1	LOC_Os04g12560	Oryza sativa	Brown planthopper (BPH) resistance gene
Abar03B063668.1	LOC_Os06g41850	Oryza sativa	1000-grain weight
Abar03B066719.1	LOC_Os08g32870	Oryza sativa	Aroma gene
Abar03B067365.1	TraesCS4D02G198600	Triticum aestivum	ABA, Drought tolerance
Abar03B067366.1	TraesCS2B02G405700	Triticum aestivum	Grain number, grain weight
Abar03B067369.1	TraesCS6A02G308600	Triticum aestivum	Tiller number,spikelet number,grain number per panicle,1000-grain weight
Abar03B067646.1	TraesCS4D02G198600	Triticum aestivum	ABA, Drought tolerance
Abar03B068141.1	TraesCS4A02G219700	Triticum aestivum	Drought tolerance
Abar03B068227.1	LOC_Os10g31850	Oryza sativa	Draught resistant gene
Abar03B068393.1	LOC_Os10g40600	Oryza sativa	Nitrate use
Abar03B068394.1	LOC_Os10g40600	Oryza sativa	Nitrate use
Abar03B068422.1	LOC_Os06g10990	Oryza sativa	Major regulator of the reproductive barrier and compatibility
Abar04B089765.1	TraesCS5D02G486600	Triticum aestivum	Confers the free-threshing character,other agronomically important traits,plant height
Abar04B093861.1	TraesCS4B02G020300	Triticum aestivum	Heading date, flowering times
Abar04B096235.1	LOC_Os10g35640	Oryza sativa	Fertility restoration gene
Abar05B021149.1	TraesCS4B02G020300	Triticum aestivum	Heading date, flowering times
Abar05B021513.1	LOC_Os04g12560	Oryza sativa	Brown planthopper (BPH) resistance gene
Abar05B021896.1	LOC_Os10g32600	Oryza sativa	Controlled flowering
Abar05B022241.1	LOC_Os12g43440	Oryza sativa	Tolerance to nitrogen deficiency
Abar05B022243.1	LOC_Os04g04330	Oryza sativa	Improve cold tolerance
Abar05B022307.1	LOC_Os06g29810	Oryza sativa	Resistance to rice blast
Abar05B024653.1	LOC_Os12g29220	Oryza sativa	Response to drought and salt stress, expand the range of resistance against *Xanthomonas oryzae* pv
Abar05B024654.1	LOC_Os11g31190	Oryza sativa	Grain filling
Abar05B024695.1	LOC_Os06g29810	Oryza sativa	Resistance to rice blast
Abar06B075419.1	LOC_Os06g16370	Oryza sativa	Control heading date
Abar06B075576.1	LOC_Os11g11790	Oryza sativa	Magnaporthe grisea resistance-a
Abar06B078116.1	LOC_Os03g62480	Oryza sativa	Enhance UV-B tolerance, modulates flavonoid metabolism
Abar06B078258.1	LOC_Os06g29810	Oryza sativa	Resistance to rice blast
Abar06B078349.1	LOC_Os04g12560	Oryza sativa	Brown planthopper (BPH) resistance gene
Abar07B0100687.1	TraesCS4B02G299400	Triticum aestivum	Root length, plant height
Abar07B0101448.1	LOC_Os11g47210	Oryza sativa	Conferring resistance for rice bacterial blight
Abar07B0101488.1	LOC_Os10g32600	Oryza sativa	Controlled flowering
Abar07B0102196.1	TraesCS1D02G254500	Triticum aestivum	Plant height and coleoptile growth
Abar07B0102875.1	TraesCS2B02G343600	Triticum aestivum	Total seed proteins, plant height, heading date, seed size, thousand kernel weight
Abar07B0103282.1	LOC_Os06g29810	Oryza sativa	Resistance to rice blast
Abar07B0104067.1	LOC_Os08g32870	Oryza sativa	Aroma gene
Abar07B0104080.1	TraesCS1A02G018600	Triticum aestivum	Leaf rust resistance
Abar07B0104088.1	LOC_Os01g44260	Oryza sativa	Adjust seed coat color
Abar07B0104094.1	TraesCS5D02G486600	Triticum aestivum	Confers the free-threshing character, other agronomically important traits, plant height
Abar07B0104098.1	LOC_Os06g29810	Oryza sativa	Resistance to rice blast
Abar07B0104118.1	LOC_Os04g04330	Oryza sativa	Improve cold tolerance
Abar07B0104126.1	LOC_Os04g04330	Oryza sativa	Improve cold tolerance
Abar07B0104198.1	TraesCS4D02G198600	Triticum aestivum	ABA,Drought tolerance
Abar07B0104204.1	LOC_Os06g29810	Oryza sativa	Resistance to rice blast
Abar07B0104218.1	LOC_Os01g10110	Oryza sativa	improve the panicle architecture and enhance the grain yield
Abar07B0105671.1	LOC_Os07g41240	Oryza sativa	seed size, grain yield
Abar07B0106358.1	TraesCS2B02G491400	Triticum aestivum	PPO activity/end-use quality/wheat products colour
Abar07B0107287.1	LOC_Os06g29810	Oryza sativa	Resistance to rice blast
Abar07B0107673.1	TraesCS4B02G020300	Triticum aestivum	heading date, flowering times
Abar07B0107783.1	LOC_Os11g35500	Oryza sativa	Bacterial blight resistance gene
Abar07B0108309.1	TraesCS1A02G018600	Triticum aestivum	Leaf rust resistance
Abar07B0108734.1	LOC_Os06g29810	Oryza sativa	Resistance to rice blast
Abar07B0109065.1	LOC_Os04g04330	Oryza sativa	Improve cold tolerance
Abar07B0109339.1	LOC_Os09g29820	Oryza sativa	Controls rice cold tolerance

Supplementary Table S14. GBS data information of 211 *A. barbata* accessions.

Name	Type	Lat	Long	Experiment Accession
DLO.27	M	40.783	-123.335	SRX16241379
SAR.23	M	36.012	-120.924	SRX16241685
PJC.08	M	40.681	-122.352	SRX16241558
CHP.37	M	38.585	-122.57	SRX16241324
LIK.33	M	37.329	-121.681	SRX16241399
RED.27	M	40.613	-122.355	SRX16241681
LIK.02	M	37.329	-121.681	SRX16241546
COM.22	M	39.269	-123.643	SRX16241720
RED.19	M	40.613	-122.355	SRX16241344
CHI.39	M	38.501	-122.353	SRX16241694
RED.39	M	40.613	-122.355	SRX16241561
LIK.17	M	37.329	-121.681	SRX16241511
BOD.01	M	38.32	-123.035	SRX13548344
COM.13	M	39.269	-123.643	SRX16241719
SLR.31	M	33.278	-117.222	SRX16241698
RED.23	M	40.613	-122.355	SRX16241680
CDR.42	M	38.578	-122.529	SRX16241607
PYN.10	M	40.338	-121.914	SRX16241343
LHD.07	M	40.888	-122.384	SRX16241500
FID.05	M	38.487	-120.805	SRX16241518
SAR.02	X	36.012	-120.924	SRX16241682
PIN.13	X	36.488	-121.153	SRX16241340
SUN.04	X	33.697	-117.177	SRX16241702
SBR.06	X	34.184	-117.329	SRX16241688
SFN.27	X	34.358	-118.555	SRX16241693
SUN.05	X	33.697	-117.177	SRX16241703
SUN.06	X	33.697	-117.177	SRX16241704
SUN.07	X	33.697	-117.177	SRX16241367
MBU.35	X	34.041	-118.892	SRX16241326
SMV.17	X	39.208	-121.256	SRX16241699
SUN.08	X	33.697	-117.177	SRX16241414
OMD.03	X	32.841	-117.043	SRX16241677
MBU.41	X	34.041	-118.892	SRX16241327
CUY.11	X	35.07	-119.99	SRX16241382
STO.12	X	39.659	-122.526	SRX16241364
MUG.03	X	34.123	-119.09	SRX16241331
MIL.17	X	37.028	-119.702	SRX16241329
SLR.05	X	33.278	-117.222	SRX16241355
ETO.04	X	33.66	-117.657	SRX16241530
LMN.11	X	36.406	-119.055	SRX16241401
KRN.09	X	35.467	-118.755	SRX16241477
ETO.01	X	33.66	-117.657	SRX16241389
PFR.15	X	36.816	-119.385	SRX16241339

ISB.10	X	35.643	-118.463	SRX16241464
COA.20	X	36.101	-120.416	SRX16241346
BSR.12	X	36.214	-121.747	SRX16241710
STO.42	X	39.659	-122.526	SRX16241413
SFN.18	X	34.358	-118.555	SRX16241409
MBU.26	X	34.041	-118.892	SRX16241325
POR.13	X	36.026	-118.922	SRX16241341
EDS.01	X	34.941	-118.925	SRX16241385
REF.10	X	34.563	-120.091	SRX16241402
SCL.10	X	33.428	-117.61	SRX16241349
OMD.34	X	32.841	-117.043	SRX16241554
SNE.15	X	37.517	-120.448	SRX16241360
LUC.24	X	35.999	-121.47	SRX16241661
OMD.31	X	32.841	-117.043	SRX16241553
GOR.10	X	34.794	-118.842	SRX16241394
COA.29	X	36.101	-120.416	SRX16241718
STO.07	X	39.659	-122.526	SRX16241701
ETO.15	X	33.66	-117.657	SRX16241531
SFN.40	X	34.358	-118.555	SRX16241351
BER.03	X	38.499	-122.121	SRX16241708
ETO.12	X	33.66	-117.657	SRX16241390
CAS.01	X	34.507	-118.603	SRX16241415
COA.31	X	36.101	-120.416	SRX16241357
ETO.18	X	33.66	-117.657	SRX16241392
LYT.11	X	34.261	-117.499	SRX16241547
SFN.03	X	34.358	-118.555	SRX16241350
MBU.18	X	34.041	-118.892	SRX16241549
SMO.03	X	34.051	-118.53	SRX16241358
OMD.21	X	32.841	-117.043	SRX16241337
CLR.08	X	39.042	-122.341	SRX16241335
SAR.16	X	36.012	-120.924	SRX16241684
SGB.06	X	34.159	-117.909	SRX16241352
CAL.11	X	35.281	-118.624	SRX16241374
LAL.23	X	34.78	-120.315	SRX16241638
COA.03	X	36.101	-120.416	SRX16241717
PAT.10	X	37.457	-121.191	SRX16241557
MPS.06	X	37.46	-119.943	SRX16241674
COA.44	X	36.101	-120.416	SRX16241368
SFN.38	X	34.358	-118.555	SRX16241695
MBU.08	X	34.041	-118.892	SRX16241548
CDR.37	X	38.578	-122.529	SRX16241672
OMD.08	X	32.841	-117.043	SRX16241678
SBA.01	X	34.462	-119.771	SRX16241406
LCS.05	X	34.41	-119.366	SRX16241649
DMR.08	X	32.931	-117.237	SRX16241387
SLR.17	X	33.278	-117.222	SRX16241697

SLR.14	X	33.278	-117.222	SRX16241696
ARC.11	MX	40.903	-124.072	SRX13548201
BEL.22	MX	37.013	-121.348	SRX13548474
BER.11	MX	38.499	-122.121	SRX16241709
BER.27	MX	38.499	-122.121	SRX16241418
BER.40	MX	38.499	-122.121	SRX16241371
BER.46	MX	38.499	-122.121	SRX16241372
BLF.17	MX	40.421	-122.191	SRX13548333
BOD.16	MX	38.32	-123.035	SRX13548355
BOD.29	MX	38.32	-123.035	SRX13548246
BOD.44	MX	38.32	-123.035	SRX13548257
BOD.53	MX	38.32	-123.035	SRX16241373
CDR.01	MX	38.578	-122.529	SRX16241606
CDR.02	MX	38.578	-122.529	SRX16241376
CDR.13	MX	38.578	-122.529	SRX16241625
CDR.26	MX	38.578	-122.529	SRX16241377
CDR.49	MX	38.578	-122.529	SRX16241683
CHI.03	MX	38.501	-122.353	SRX16241711
CHI.15	MX	38.501	-122.353	SRX16241712
CHI.29	MX	38.501	-122.353	SRX16241713
CHI.48	MX	38.501	-122.353	SRX16241378
CHO.14	MX	35.711	-120.31	SRX16241714
CHP.02	MX	38.585	-122.57	SRX16241705
CHP.17	MX	38.585	-122.57	SRX16241608
CHP.18	MX	38.585	-122.57	SRX16241716
CHP.22	MX	38.585	-122.57	SRX16241391
CHP.49	MX	38.585	-122.57	SRX16241715
COM.11	MX	39.269	-123.643	SRX16241380
COM.37	MX	39.269	-123.643	SRX16241721
COM.41	MX	39.269	-123.643	SRX16241381
DAL.24	MX	40.312	-122.007	SRX16241383
DLO.01	MX	40.783	-123.335	SRX16241386
DLO.08	MX	40.783	-123.335	SRX16241384
DLO.35	MX	40.783	-123.335	SRX16241534
DLO.50	MX	40.783	-123.335	SRX16241517
ELK.14	MX	39.61	-122.532	SRX16241388
FID.08	MX	38.487	-120.805	SRX16241532
FID.15	MX	38.487	-120.805	SRX16241533
FID.28	MX	38.487	-120.805	SRX16241535
FID.30	MX	38.487	-120.805	SRX16241545
FID.44	MX	38.487	-120.805	SRX16241556
FOR.08	MX	40.6	-124.17	SRX16241536
GEY.01	MX	38.712	-122.882	SRX16241393
GEY.12	MX	38.712	-122.882	SRX16241537
GEY.19	MX	38.712	-122.882	SRX16241417
GEY.27	MX	38.712	-122.882	SRX16241538

GEY.44	MX	38.712	-122.882	SRX16241609
GEY.45	MX	38.712	-122.882	SRX16241539
GEY.60	MX	38.712	-122.882	SRX16241407
GEY.75	MX	38.712	-122.882	SRX16241420
GUA.10	MX	38.785	-123.554	SRX16241519
HIX.11	MX	38.131	-122.713	SRX16241430
HKR.11	MX	40.293	-122.277	SRX16241569
HOP.18	MX	39.007	-123.08	SRX16241580
HOP.50	MX	39.007	-123.08	SRX16241395
HOP.61	MX	39.007	-123.08	SRX16241540
HOP.72	MX	39.007	-123.08	SRX16241591
HOP.90	MX	39.007	-123.08	SRX16241442
ING.10	MX	40.756	-122.026	SRX16241453
JAK.16	MX	38.36	-120.745	SRX16241541
JCT.08	MX	40.725	-123.05	SRX16241396
JEN.06	MX	38.498	-123.208	SRX16241397
JEN.10	MX	38.498	-123.208	SRX16241542
JEN.14	MX	38.498	-123.208	SRX16241603
JEN.29	MX	38.498	-123.208	SRX16241614
JEN.33	MX	38.498	-123.208	SRX16241466
JOL.14	MX	35.963	-121.185	SRX16241398
LAL.02	MX	34.78	-120.315	SRX16241488
LAL.07	MX	34.78	-120.315	SRX16241627
LAL.34	MX	34.78	-120.315	SRX16241543
LAL.41	MX	34.78	-120.315	SRX16241544
LIK.01	MX	37.329	-121.681	SRX16241421
LIK.11	MX	37.329	-121.681	SRX16241610
LIK.37	MX	37.329	-121.681	SRX16241400
LIK.71	MX	37.329	-121.681	SRX16241522
MEN.11	MX	39.24	-123.181	SRX16241328
MOS.17	MX	37.528	-122.513	SRX16241673
MQR.02	MX	35.555	-120.888	SRX16241330
MSH.04	MX	38.18	-122.909	SRX16241550
MSH.11	MX	38.18	-122.909	SRX16241675
MSH.22	MX	38.18	-122.909	SRX16241676
MSH.31	MX	38.18	-122.909	SRX16241551
MSH.43	MX	38.18	-122.909	SRX16241552
NAC.03	MX	35.998	-121.383	SRX16241332
NAP.07	MX	38.209	-122.187	SRX16241333
NAV.10	MX	39.111	-123.507	SRX16241334
OAK.20	MX	40.652	-122.599	SRX16241336
ORO.21	MX	39.518	-121.513	SRX16241679
PAI.01	MX	36.681	-121.259	SRX16241338
PAS.18	MX	39.851	-122.615	SRX16241555
PRB.01	MX	35.589	-120.7	SRX16241559
PST.09	MX	36.19	-120.706	SRX16241342

RBF.23	MX	40.215	-122.178	SRX16241560
RED.34	MX	40.613	-122.355	SRX16241345
RIC.01	MX	40.032	-123.784	SRX16241403
SAD.16	MX	39.183	-123.754	SRX16241520
SAN.01	MX	38.218	-120.69	SRX16241404
SAR.30	MX	36.012	-120.924	SRX16241687
SAR.36	MX	36.012	-120.924	SRX16241348
SAU.16	MX	37.836	-122.488	SRX16241405
SFH.02	MX	39.234	-121.295	SRX16241689
SFH.16	MX	39.234	-121.295	SRX16241690
SFH.17	MX	39.234	-121.295	SRX16241691
SFH.34	MX	39.234	-121.295	SRX16241408
SFH.46	MX	39.234	-121.295	SRX16241692
SIL.28	MX	38.345	-122.283	SRX16241353
SJB.22	MX	36.853	-121.569	SRX16241354
SLD.09	MX	37.097	-121.121	SRX16241410
SLR.27	MX	33.278	-117.222	SRX16241356
SMR.16	MX	41.844	-124.018	SRX16241411
SMT.10	MX	37.494	-122.371	SRX16241359
SNL.21	MX	37.601	-121.87	SRX16241700
SOL.04	MX	36.433	-121.365	SRX16241361
SON.11	MX	38.237	-122.514	SRX16241412
SPR.21	MX	36.572	-121.741	SRX16241362
SSM.14	MX	35.646	-121.192	SRX16241363
STO.24	MX	39.659	-122.526	SRX16241521
STO.27	MX	39.659	-122.526	SRX16241366
UKH.10	MX	39.111	-123.197	SRX16241369
WEO.06	MX	40.322	-123.921	SRX16241370
WLT.07	MX	39.521	-123.393	SRX16241706
WOD.11	MX	35.714	-118.86	SRX16241707

Supplementary Table S15. F3-Statistics result.

F3 (Target;Source1, Source2)	F3 statistic	Standard error	Z_score
mesic; xeric, MX	0.0211798	0.0010326	20.5112
MX; xeric, mesic	-0.00279262	0.000617105	-4.52536
xeric; mesic, MX	0.031594	0.00161154	19.6049

Supplementary Table S16. Functional annotation of genes containing SNPs that distinguish between two ecotypes.

Gene	Description
Abar01A080553.1	RING-H2 finger protein ATL63-like
Abar01A080578.1	disease resistance protein Pik-2-like
Abar01A083596.1	cytochrome P450 72A11-like
Abar01A086941.1	3-ketoacyl-CoA synthase 6
Abar01B011298.1	pentatricopeptide repeat-containing protein At4g32430, mitochondrial
Abar02A060308.1	transcription factor MYB41-like
Abar03A01881.1	protein SUBSTANDARD STARCH GRAIN 4, chloroplastic
Abar03A0929.1	G-type lectin S-receptor-like serine/threonine-protein kinase SD2-5
Abar03B071735.1	protein NETWORKED 2D-like
Abar04A0126117.1	probable inactive leucine-rich repeat receptor-like protein kinase At3g03770 isoform X2
Abar04A0126469.1	F-box protein
Abar04A0126469.1	F-box protein At1g47340-like
Abar04A0128581.1	protein indeterminate-domain 14-like
Abar04B097884.1	F-box/FBD/LRR-repeat protein
Abar05A046303.1	peroxidase 5-like
Abar05A049850.1	hypothetical protein GOBAR
Abar05B026424.1	protein indeterminate-domain 14-like
Abar05B026894.1	IRK-interacting protein-like
Abar05B027885.1	RING-H2 finger protein
Abar05B028227.1	transcription factor MYC2-like
Abar06A042356.1	ent-kaur-16-ene synthase, chloroplastic-like
Abar06A042513.1	pentatricopeptide repeat-containing protein At4g33990-like
Abar07A0110212.1	peroxisomal membrane protein 11-5
Abar07A0110854.1	putative multidrug resistance protein
Abar07A0112828.1	pathogenesis-related thaumatin-like protein 3.5
Abar07A0118129.1	MADS-box transcription factor 16
Abar07B0104972.1	trehalose-6-phosphate phosphatase1
Abar07B0109601.1	disease resistance protein At4g27190-like

Supplementary Table S17. Primer list for the qRT-PCR analysis.

Gene	Forward primer (5′-3′)	Reverse primer (5′-3′)
Abar01A083459.1	GCATGGAGGCGTACAACAAG	GCCTGACTTTGTCTCCTGCT
Abar01A083610.1	ATGGCCGGGACTATGACTCT	TCTTGGGCAGGAACGACTTC
Abar01A086680.1	ACCATCGCCATCAGCTTGG	CCAGAACTCGTCGAAGCCC
Abar01A086757.1	TAAGGAGACCGTAGTGCCGA	GTGGCTGCTTACACCTGACT
Abar01B010894.1	CAGAAGTCCCACTCCAACCC	AGCTGATGAGGTTTGCACCA
Abar04B094850.1	CTCAGGGAGGCAAGCAATCA	AAGTTGGCACGACAGCACTA
Abar04A0126469.1	TACTGAACCTGGACCGCCTA	GACAACCAGACCACGAGGAG
Abar04A0128016.1	GGAACTGCGAGCTACATCCA	ATCGAAAACTTCGGCGGTCT
Abar03B071202.1	CAGGTCAGCCTTCTGAGCAA	CCCTTCGTTCTCCAGCACTT
Abar03B071204.1	CAACCACAACGCTTACGAGC	AGCCGTGTTCTCCTTCATGG
Abar05B025588.1	CTACAACGGCGATCACACCT	ATCTGCCCGAAGCAGTTCTC
Abar06A039496.1	CCTCGGTATGCTCGGATGAC	ATCCGCCTGACTTCTTCTGC
Abar07B0100325.1	CACTACAACCCCTTCGTGCT	GGCTCTCGTCCTTGATCTCG
Abar07B0109930.1	GTCATGAACGAGTACCGCCT	CTTCCGGTACACCTTGCACA
Abar07A0110212.1	GAAGTCCAACGAGAGGCTCC	GTAACAAGCGATGAGCGAGC
Abar04A0128581.1	CTACAAGGCGCACCTCAAGA	GTGTCCTGGTGCTCGATGAA
Abar03B064686.1	GGCTTCAGTAGGCGTAGCTC	TCAGGCAACTTCTCAACGCC
Abar03A02986.1	ATGGCTTCAGTAGGCGTAGC	AACTTCTCAACACCCACGCT
San03A02470.1	GTAGGCATAGCTCCGTCAGG	GATCAGGCAACTTCTCAACGC
San03D024158.1	ATGGCTTCAGTAGGCGTAGC	GATCAGGCAACTTCTCAACGC
